# Ferroptosis with Contributions from Apoptosis and Necroptosis in Porphyrazine III-Based Photodynamic Therapy of Primary Human Gliomas

**DOI:** 10.3390/pharmaceutics18060705

**Published:** 2026-06-08

**Authors:** Ekaterina Sleptsova, Alina Khuzina, Daria Sachkova, Diana Yuzhakova, Yevgeniya Sannova, Konstantin Yashin, Nina Peskova, Svetlana Lermontova, Ilya Shchechkin, Larisa Klapshina, Irina Balalaeva, Victoria Turubanova

**Affiliations:** 1 Department of Genetics and Life Sciences, Sirius University, 354340 Sochi, Russia; ees222@list.ru (E.S.); huzinaar@gmail.com (A.K.);; 2 Institute of Experimental Oncology and Biomedical Technologies, Privolzhsky Research Medical University, 603005 Nizhny Novgorod, Russia; sachkova.collins@gmail.com (D.S.); yuzhakova-diana@mail.ru (D.Y.);; 3 Institute of Biology and Biomedicine, Lobachevsky State University, 603022 Nizhny Novgorod, Russia; nin-22@yandex.ru (N.P.);; 4 Department of Neurosurgery, Privolzsky Research Medical University, 603005 Nizhny Novgorod, Russia; 5 Sector of Chromophors for Medicine, G.A. Razuvaev Institute of Organometallic Chemistry of the Russian Academy of Sciences, 603137 Nizhny Novgorod, Russia; 6 Institute of Information Technology, Mathematics and Mechanics, Lobachevsky State University, 603022 Nizhny Novgorod, Russia; 7 Institute of Neurosciences, Lobachevsky State University, 603022 Nizhny Novgorod, Russia

**Keywords:** ICD, PDT, ferroptosis, necroptosis, apoptosis, reactive oxygen species, ER, Golgi, mitochondrial membrane potential

## Abstract

**Background**: Photodynamic therapy (PDT) leading to immunogenic cell death (ICD) may serve as a promising basis for the development of antitumor therapeutic strategies. However, the mechanisms of action of photoinduced ICD in primary tumor cultures, including human glioma, remain unexplored. **Methods**: In the present study, the features of regulated cell death induced by photodynamic therapy using a previously described ICD inducer, porphyrazine III (pz III), were investigated. Cell death was studied in 7 primary cultures of high-grade human gliomas (astrocytomas, oligodendrogliomas, and glioblastomas). **Results**: Accumulation of porphyrazine III was observed in the endoplasmic reticulum (ER), Golgi apparatus, lysosomes, and mitochondria; however, the distribution of the photosensitizer varied across different cultures. A narrow concentration window of porphyrazine III was established to effectively reach IC85, primarily inducing ferroptosis with contributions from apoptosis and necroptosis accompanied by superoxide anion generation and mitochondrial dysfunction. **Conclusions**: Given the immunogenic potential of ferroptosis, apoptosis and necroptosis we hypothesize that the induction of PDT using porphyrazine III in glioma will trigger immunogenic cell death.

## 1. Introduction

Photodynamic cell death is initiated by the activation of a photosensitizer upon exposure to light of an appropriate wavelength, which leads to intensive generation of reactive oxygen species (ROS) and the development of oxidative damage to key subcellular compartments—mitochondria, lysosomes, the Golgi apparatus (GA), and the endoplasmic reticulum (ER) [1]. Induced oxidative stress initiates one or several signaling pathways of regulated cell death (including apoptosis, necroptosis, ferroptosis, and other forms thereof), the choice of which is determined by the type of photosensitizer, the subcellular localization of the photosensitizer, and the dose [2,3,4,5,6,7]. PDT is a promising method; however, its application is either largely limited to superficial tumors or requires high-technology delivery approaches [8]. Nevertheless, we hypothesize that PDT can be used as a weapon that induces cell death and leads to favorable immunological consequences.

Gliomas represent a heterogeneous group of malignant neoplasms of the central nervous system characterized by infiltrative growth. By penetrating the surrounding healthy brain tissue, they make complete surgical resection impossible without a substantial risk of damaging functionally significant areas. High genetic heterogeneity, the presence of a cancer stem cell population, and pronounced adaptive plasticity are typical for gliomas, particularly for the highly aggressive glioblastoma (GBM) [9,10]. These biological features underlie resistance to standard therapeutic modalities, including radiotherapy and chemotherapy [11,12,13]. A significant limiting factor is the blood–brain barrier, which substantially impedes the delivery of chemotherapeutic agents to the central nervous system. This restricted access largely explains the poor prognosis associated with gliomas, which are characterized by one of the lowest five-year survival rates in oncology. Remarkably, even with intensive combination therapy, the median overall survival of patients with the most aggressive form—glioblastoma—rarely exceeds 12–15 months [14,15,16]. This high mortality rate associated with the disease underscores the urgent need for the development of novel and effective therapeutic approaches.

We have previously presented data on the development of novel approaches to cellular immunotherapy using murine models of glioma [17,18,19]. Additionally, the mechanisms of cell death induced by PDT are being studied in various glioma models [20,21,22,23,24]. However, the transition to translational research in this complex area of oncology is critically necessary [8,25,26]. Therefore, we continue our research using primary cultures of human brain tumors, which represent a more relevant model.

Immunotherapeutic approaches based on photo-induced immunogenic cell death (ICD) hold great promise [27]. Immunogenic cell death is the demise of tumor cells that is accompanied by the active release of DAMPs (Damage-associated molecular patterns), as well as the establishment of antitumor adaptive immunity.

ICD was first described by Guido Kroemer in 2005 as immunogenic apoptosis [28]. Immunogenic cell death is a regulated form of cell death (including apoptosis, necroptosis, and other stress-induced types) that is accompanied by the release of damage-associated molecular patterns [29,30]. DAMPs endow dying cells with the ability to induce immunological memory. ICD confers upon dying cancer cells the capacity to function as an “anticancer vaccine,” which provides for the activation of dendritic cells and the cross-presentation of antigens [31,32]. Immunogenically dying cells are characterized by their ability, in immunocompetent syngeneic hosts, to elicit antigen-specific immune responses comprising both an effector phase and the establishment of immunological memory. This is demonstrated in vivo by vaccinating immunocompetent mice with cancer cells subjected to ICD induction, followed by injection of viable tumor cells of the same type, and assessment of tumor growth control, protection against rechallenge, and the formation of long-term antigen-specific immunological memory [33,34,35,36,37].

**Figure 1:**
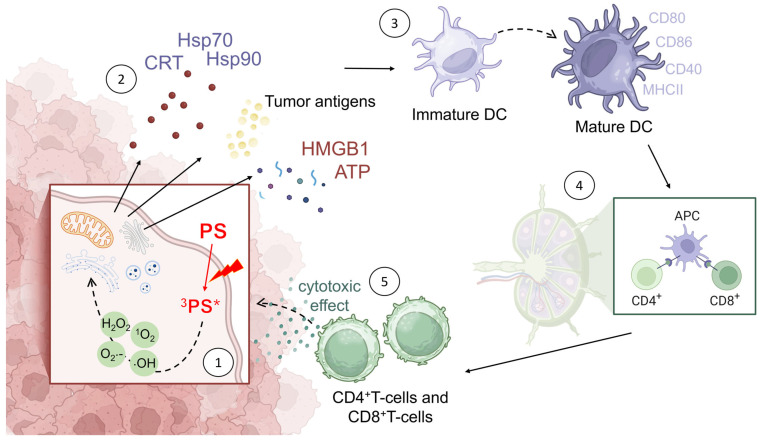
Antitumor efficacy of photoinduced immunogenic cell death. (1) A photosensitizer (PS) accumulating within the cell is photoactivated by light and generates ROS at the site of accumulation, which promote cell death. Under a specific combination of conditions, regulated cell death occurs. (2) If RCD is accompanied by the release of DAMPs as adjuvants (exposure of CRT, Hsp70, Hsp90; release of HMGB1, ATP), the death may be classified as ICD. (3) During ICD, dendritic cells (DCs) phagocytose tumor antigens and become activated in response to DAMP signals. (4) Activated DCs then migrate to the lymph nodes and present tumor antigens to T-cell immunity. (5) Activation of the adaptive arm of the immune system leads to a robust cytotoxic response against tumor antigens and also facilitates the establishment of immunological memory. APC—antigen-presenting cells, PS—photosensitizer, DC—dendritic cell.

Photodynamic therapy has been repeatedly described in the literature as an inducer of immunogenic cell death, particularly in cases where it elicits appropriate emission of DAMPs and cross-presentation of tumor antigens [17,38,39,40]. Photoinduced immunogenic cell death has been described in a number of studies using tumor cell lines; however, there is a lack of research on the efficacy of ICD inducers in primary tumor cultures. Due to the complexity and heterogeneity of patient gliomas, a challenge remains to properly direct cell death toward a regulated form in order to achieve ICD (Figure 1).

In previous works, tetra(aryl)tetracyanoporphyrazine with a pendant [4-(4-fluorobenzyloxy)phenyl] group (hereinafter referred to as porphyrazine III, pz III in figures) [41] was described as an inducer of immunogenic cell death, accompanied by the release of DAMPs from dying tumor cells and the formation of adaptive immunity in vaccinated mice [19,42]. Tetra(aryl)tetracyanoporphyrazines are readily soluble in media, selectively accumulate in tumor cells, and act as environmental viscosity sensors (upon entering a viscous environment, the photosensitizer begins to ‘light up’, allowing easy detection). Moreover, while exhibiting consistently high efficiency under photoirradiation, the porphyrazine has low dark toxicity. However, we studied its toxic effects on primary neuron-glia cultures from mice and arrived at the unambiguous conclusion that in vivo use of PDT is impossible due to significant damage to normal brain cells [43].

In this work, we are seeking an approach to properly kill patient tumor cells in a manner that ultimately leads to a regulated form of cell death. We are systematically studying the photochemical effects exerted on cells by tetra(aryl)tetracyanoporphyrazine in combination with irradiation at a dose of 20 J/cm^2^. It is important for us to monitor cell death at all stages in order to select conditions under which cells would die gradually yet effectively, without progressing to necrosis. We also assess the type of cell death and analyze how it depends on the localization of the photosensitizer and on which reactive oxygen species this photosensitizer induces within the cell. Ultimately, we demonstrate the conditions that lead to RCD of glioma cells.

## 2. Materials and Methods

### 2.1. Glioma Cell Culture

The study used 7 primary cultures of human glioma. The primary glioma cell cultures have been established in the Clinical Center of Privolzhsky Research Medical University. Tumor specimens were obtained from patients during tumor resection. Informed written consent was obtained from each patient prior to enrollment. This study was approved by the local human research ethics committee of Privolzhsky Research Medical University (protocol #7 from 20 Jun 2025). Glioma cells were isolated from patient resection material according to reference [43]. Glioma cells were cultured at 37 °C under 5% CO_2_ in RPMI-1640. The medium containing 4.5 g/L glucose and supplemented with 2 mM glutamine, 100 μM sodium pyruvate, 100 units/mL penicillin, 100 μg/L streptomycin and 10% fetal bovine serum. Given the high interpatient heterogeneity, we selected individual culture protocols for each culture: the seeding density and passage frequency, trypsin incubation time, and centrifugation conditions were variable. Cultures were regularly tested for mycoplasma using the MycoReport kit (Evrogen, Russia, Moscow). Cell cultures were retrieved from cryopreservation (at passage 1–2 after the establishment of a 2D cell layer on culture plastic) and were subjected to cryopreservation in RPMI-1640 medium supplemented with 30% fetal bovine serum and 8% DMSO no later than passage 3 in order to preserve the properties of primary cells and maintain heterogeneity. Phase-contrast images of primary cultures derived from patient gliomas were obtained at passages 3–4, at least 24 h after subculturing, using the inverted EVOS M5000 Imaging System (Invitrogen, Thermo Fisher Scientific, Waltham, MA, USA) at 20× magnification.

### 2.2. Induction of Cell Death by Porphyrazine III-Based PDT

Cell death was induced by photodynamic treatment using tetra(aryl)tetracyanoporphyrazine bearing a 4-(4-fluorobenzyloxy)phenyl group in the aryl frame of the macrocycle (patent RU2672806C1, Russia; Supplementary Figure S1). The absorbance and fluorescence spectra of porphyrazine III, relevant for PDT, have been characterized previously (see Figure 1C therein [42]). For photodynamic treatment, cells were loaded with the photosensitizer for 4 h in serum-free culture medium. Subsequently, glioma cells were irradiated in full culture medium without porphyrazine III for 16 min and 40 s to achieve a dose of 20 J/cm^2^ using an LED light source (λex = 630 nm). The irradiation dose was delivered at a constant power density of 32 mW/cm^2^. Beam uniformity is ensured by a dedicated emitter, which exposes the entire cell surface to the same dose. Furthermore, irradiation is performed using a heating stage set to 35 °C, which eliminates photothermal effects on the cells and maintains a physiological temperature during exposure. After irradiation, the cells were incubated in the dark in a CO_2_ incubator until further analysis.

For the photosensitizer pz III, the following photophysical parameters were previously determined: absorption maximum at 590 nm, fluorescence maximum at 642 nm, molar extinction coefficient (ε) of 2.1 × 10^4^ L × mol^−1^ × cm^−1^, and fluorescence quantum yield (Φf) of 0.1%. Regarding photobleaching kinetics, an overall assessment of photostability was performed in previous studies: at a light dose of 100 J/cm^2^ (conditions close to those used in our experiments), the degree of photobleaching was approximately 50%, indicating acceptable stability of the compound [42,44]. The evaluation of the singlet oxygen generation quantum yield (ΦΔ) for pz III was the subject of a separate study [45].

### 2.3. Cellular Uptake and Subcellular Distribution of Porphyrazine

The accumulation dynamics and subcellular localization were evaluated using an LSM 710 Axio Observer Z1 DUO NLO laser scanning microscope (Carl Zeiss, Jena, Germany) or an LSM 980 with Airyscan 2 laser scanning microscope (Carl Zeiss, Jena, Germany). Glioma cells were seeded overnight at a density of 2 × 10^4^ cells per well in 96-well glass-bottom plates. To assess the accumulation level, wash-free monitoring was performed: after adding a medium with photosensitizers at a concentration of 3 µM, images of cells were obtained every hour for 4 h. Porphyrazine III fluorescence was recorded in the range of 600–670 nm when excited at 594 nm or 630 nm. For semiquantitative analysis, the fluorescence intensity of the cytoplasmic region of the cells was measured using ZEN 2.6. 2018 software (Carl Zeiss Microscopy GmbH, Oberkochen, Germany); at least 20 cells in three fields of view were analyzed.

To determine subcellular localization, the medium was replaced with a serum-free medium containing 3 µM of the photosensitizer under study, and the cells were incubated for 4 h. The following dyes were added for 30 min according to the manufacturer’s instructions: 0.5 μM LysoTracker Green DND-26 for lysosomes, 0.5 μM ER-Tracker for the endoplasmic reticulum (ER), 0.5 μM MitoTracker Green FM for mitochondria, 5 μM BODIPY FL C5-ceramide complexed with BSA for the Golgi apparatus (GA), and 1.0 μM Hoechst for the nucleus. Excitation of LysoTracker, ER-Tracker, MitoTracker and BODIPY FL was done by laser at 488 nm and fluorescence was registered in the range of 500–560 nm; excitation of Hoechst was done by laser at 405 nm and fluorescence was registered in the range of 430–470 nm.

To assess colocalization of signals from two channels in confocal images acquired using Zeiss Zen software (Zen 2.6, Carl Zeiss Microscopy GmbH, Oberkochen, Germany), intensity profiles were generated along selected line scans (X-axis: distance in µm; Y-axis: intensity in arbitrary units). The resulting numerical data were analyzed in GraphPad Prism 10 (San Diego, CA, USA). Correlation analysis was performed using the Pearson’s correlation coefficient. Statistical significance was determined by *p* < 0.05 and a 95% confidence interval for r excluding zero; correlation strength was interpreted on a scale where >0.7 indicated strong colocalization and 0.4–0.7 moderate colocalization.

For detailed visualization of mitochondria in glioma cells previously seeded onto 35 mm diameter glass-bottom Petri dishes and subjected to PDT, vital staining was performed using MitoTracker Green FM (Thermo Fisher Scientific, Waltham, MA, USA) for 5 min, followed immediately by image acquisition. Fluorescence recording was carried out using confocal laser scanning microscopy on an Axio Observer LSM 980 (Carl Zeiss, Jena, Germany) equipped with an Airyscan 2 super-resolution system and a system for long-term live-cell analysis within a CO_2_ incubator, under constant temperature and humidity control, without removing the cells from the controlled environment.

MitoTracker Green fluorescence was excited with a 488 nm laser and detected in the 495–550 nm range using the Airyscan system; porphyrazine fluorescence was excited with a 631 nm laser, and emission was recorded in the 650–700 nm range. Images were acquired at ×100 magnification.

Untreated glioma cells were used as a control for normal mitochondrial morphology. To assess the effect of the photosensitizer on mitochondria, imaging was also performed 4 h after co-incubation of cells with pz III.

### 2.4. Analysis of Dark Toxicity and Photodynamic Activity of Porphyrazine

To describe the photodynamic effect, it is important to understand both the direct toxicity of the photosensitizer and the toxicity of PDT using this photosensitizer.

Cells were seeded in 96-well plates at a density of 1.5–2 × 10^4^ cells per well overnight. To assess the direct toxicity of the photosensitizer, cells were incubated for 4 h in medium containing 0.1–100 μM porphyrazine III, after which the medium was replaced with complete medium, and the cell cultures were incubated for 24 h.

To evaluate the cytotoxicity of porphyrazine III-based PDT, following a 4 h photosensitizer loading at concentrations of 0.05–3.5 μM, cells were irradiated in photosensitizer-free complete medium. Cells were then incubated for 24 h and subsequently subjected to MTT analysis.

Upon data processing, viability values were calculated as percentages relative to the mean value of control live cells. Logarithmic dose–response curves were generated by transforming data in GraphPad Prism using the formula X = log(X), where X represents the photosensitizer concentration.

### 2.5. Cell Viability Analysis by MTT Assay

For the MTT assay, a 5 mg/mL solution of 3-(4,5-dimethylthiazol-2-yl)-2,5-diphenyltetrazolium bromide in DMSO was used. The cell culture medium was replaced with serum-free medium containing 10% MTT solution (final concentration 0.5 mg/mL), and the cells were incubated for 2 h. The medium was then removed, DMSO was added, and the plates were shaken for 10 min at room temperature. Optical density was measured at 570 nm using a Synergy MX spectrophotometer-fluorometer (Synergy MX microplate reader, BioTek Instruments, Winooski, VT, USA).

### 2.6. Cell Death Assay by Flow Cytometry

To select optimal conditions for inducing regulated cell death, glioma cells were treated with 1.5–2.3 μM pz III and subjected to irradiation. After 24 h, the cells were detached from the culture plastic and used for cytofluorimetric analysis.

The glioma cells were washed in Annexin V binding buffer and stained with 0.5 μM SYTOX Blue Nucleic Acid Stain (Molecular Probes) and FITC Annexin V (Invitrogen) according to the manufacturer’s instructions. The assay was performed using a CytoFLEX (Beckman Coulter, Brea, CA, USA) and a FACSAria III (BD Biosciences, Franklin Lakes, NJ, USA). The gating strategy was identical across all cultures, taking into account the autofluorescence of live cells and relative to necrotic control cells (freeze-thawed, FT).

### 2.7. Analysis of Cell Death Type

The following inhibitors were used to block cell death modalities: the pan-caspase inhibitor carbobenzoxy-valyl-alanyl-aspartyl-[O-methyl]-fluoromethylketone (z-VAD-fmk, 50 μM, Sigma-Aldrich, St. Louis, MO, USA), the RIPK1 inhibitor necrostatin-1 s (Nec-1 s, 20 μM, Abcam, Cambridge, MA, USA), and the inhibitor of ROS and lipid peroxidation ferrostatin-1 (Fer-1, 1 μM, Sigma-Aldrich, St. Louis, MO, USA) [43]. Glioma cells were seeded overnight at a density of 1.5–2 × 10^4^ cells per well in 96-well plates (depending on the cell culture). The cell death inhibitors were added together with the photosensitizer, and the cells were incubated for 4 h in serum-free conditions. Before PDT, the medium was replaced with complete medium containing the respective cell death inhibitor, the cells were irradiated with light at 20 J/cm^2^, and then they were incubated for 14 h. Assessment was performed by analyzing cell viability upon inhibition using the MTT assay.

### 2.8. Determination of the Type of Reactive Oxygen Species Produced

Glioma cells were seeded overnight at a density of 1.5–2 × 10^4^ cells per well in 96-well plates. The medium was replaced with complete medium containing 10 μM of various ROS scavengers—D-mannitol, Tiron, and sodium azide—and the cells were incubated for 20 h. After this, the medium was replaced with medium containing pz III and the scavengers for 3.5 h. Following this incubation, all medium was replaced with PBS containing 10% FBS and the nonspecific ROS scavenger 2′,7′-dichlorodihydrofluorescein diacetate (DCFH-DA), and the plate was placed in a CO_2_ incubator for another 30 min. During all these procedures, darkness was maintained in the room. After this, the initial fluorescence measurement was taken. Before measuring the fluorescence intensity, the DCFH-DA solution was replaced with PBS. Fluorescence intensity was measured using a Synergy MX microplate reader. Fluorescence was excited at 488 nm and recorded at 525 nm; a reading of each well was taken from the center of each well (read type: end point, top). Next, the cells were either exposed to irradiation at a dose of 20 J/cm^2^ or kept in the dark without irradiation. Fluorescence measurements were performed immediately after PDT, and at 15 min, 45 min, 90 min, and 120 min.

### 2.9. Analysis of the Mitochondrial Membrane Potential in Photoinduced Cells

The analysis was performed according to [46]. Cells subjected to photodynamic treatment were harvested at 45 min, 90 min, and 3 h post-irradiation. For JC-1 staining, cells were resuspended in an appropriate buffer at a 1:100 dilution and incubated for 30 min at 37 °C in the dark. Following incubation, cells were washed twice with PBS to remove excess dye, then stained with 0.5 μM SYTOX Blue nuclear dye for 10 min at room temperature to assess cell viability. Samples were analyzed by flow cytometry using a CytoFLEX (Beckman Coulter) equipped with a 488 nm blue laser (530/30 nm for JC-1 monomers [green], 585/42 nm for JC-1 aggregates [red]), a 405 nm violet laser (450/50 nm for SYTOX Blue), and a 630 nm red laser (630/30 nm for pz III). Data were acquired for at least 10,000 events per sample and analyzed using FlowJo software v10.8 (Ashland, OR, USA).

As a positive control for mitochondrial depolarization, cells were treated with hydrogen peroxide (H_2_O_2_; final concentration 1 μM, prepared fresh from 3% stock) for 1 h at 37 °C prior to JC-1 staining. Untreated cells served as negative controls. Mitochondrial membrane potential (ΔΨm) was quantified as the ratio of red (JC-1 aggregates) to green (JC-1 monomers) fluorescence intensity.

### 2.10. Redox Status of Dying Cells

Non-invasive monitoring of the redox state of human glioma cell cultures was performed using two-photon fluorescence microscopy of endogenous cofactors NAD(P)H and FAD under control conditions (no photosensitizer), 4 h post-photosensitizer addition, and at early stages of photoinduced cell death (15, 45, and 90 min, as well as 3 and 24 h post-photodynamic treatment). The dynamics of the redox state were evaluated using a Carl Zeiss LSM 880 laser scanning microscope (Carl Zeiss, Germany).

Two-photon fluorescence of NAD(P)H and FAD was excited with a Ti:Sapphire femtosecond laser MaiTai HP (Spectra-Physics Inc., Milpitas, CA, USA, repetition rate 80 MHz, pulse duration 120 fs) was used for two-photon excitation at wavelengths of 750 nm or 900 nm and registered in the ranges 455–490 nm or 500–550 nm, respectively. An oil immersion objective C Plan-Apochromat 40×/1.3 (Carl Zeiss, Jena, Germany) was used to collect the fluorescence signal. During signal collection, the cells were maintained at 37 °C and 5% CO_2_.

The fluorescence intensity of NAD(P)H and FAD was calculated by selecting the cytoplasm area of each cell on the corresponding fluorescence images using ImageJ 1.39p software (Washington, DC, USA). The redox ratio was calculated on a pixel-by-pixel by dividing NAD(P)H fluorescence intensity by FAD fluorescence intensity. The final result was corrected by subtracting the background signal, which was obtained from a cell-free area of the fluorescence image.

### 2.11. Statistical Analysis

To assess the colocalization of the photosensitizer with cellular compartment trackers, Pearson’s correlation coefficient was used, calculated based on the fluorescence intensities of pixels in two image channels from confocal microscopy using ImageJ 14.3u/Fiji software. Colocalization was considered statistically significant at r > 0.3, which corresponds to a weak positive correlation and allows discrimination of specific spatial signal overlap from random coincidence. The analysis was performed for 3 independent experiments with n ≥ 10 cells per group, and the results are presented as mean values ± SEM.

For the analysis of phototoxicity and dark toxicity data on cell viability, GraphPad Prism 10.3.1.509 software was employed. Raw data were transformed using a logarithmic scale conversion formula to fit a sigmoidal dose–response curve, which normalizes the distribution and enhances the accuracy of nonlinear regression fitting. Concentrations of the test compounds were log-transformed (log10), and dose–response inhibition curves (four-parameter variable slope model) were generated by plotting cell viability percentage (Y) against log[concentration] (X), with standard deviation error bars for each data point.

Subsequently, cells were analyzed to calculate IC_85_ values, defined as the concentration of the compound inducing 85% inhibition of viability. IC_85_ parameters, including 95% confidence intervals, were directly extrapolated from the curve-fitting results in GraphPad Prism.

Statistical analysis for the results of the inhibition assay and the NADPH/FAD ratio data was performed using the nonparametric Mann–Whitney U test to assess differences between groups. This test was chosen for the analysis of the inhibition assay results and the NAD(P)H/FAD ratio data because the data exhibited a non-normal distribution (according to the results of the D’Agostino–Pearson omnibus normality test, Shapiro–Wilk test, Kolmogorov–Smirnov test, and Anderson–Darling test, *p* < 0.05), as well as due to the presence of outliers and a relatively small sample size (n < 50).

The determination of the type of active oxygen forms was carried out using the two-way ANOVA with Tukey’s multiple comparisons test.

## 3. Results

### 3.1. Description of Glioma Cell Cultures

The study was performed on 7 human glioma cell cultures established from resected material obtained from patients of the PIMU University Clinic diagnosed with oligodendroglioma, astrocytoma, or glioblastoma. The clinical diagnosis of the patients was established according to the fifth edition of the WHO Classification of Tumours of the Central Nervous System (WHO CNS5). The clinical characteristics of the tumors from which the cell cultures were derived are presented in Table 1. Microscopic images and detailed descriptions of the cultures are provided in Appendix A.

### 3.2. Accumulation Dynamics and Subcellular Distribution in Glioma Cells

**Figure 2:**
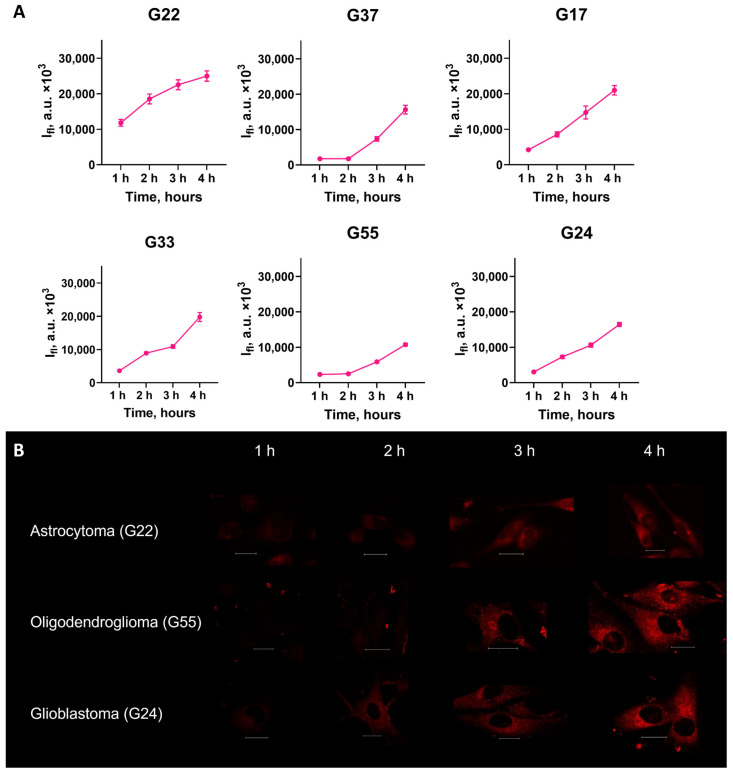
**Assessment of pz III accumulation rate in different glioma subtypes.** (**A**) Quantification of the fluorescence signal in glioma cells incubated with pz III expressed as mean ± SD (n ≥ 10). The background intensity (Ifl) of the fluorescence signal before the addition of the photosensitizer did not exceed 0.3 a.u. Data are presented for at least 10 cells from no fewer than 3 fields of view. (**B**) The uptake of Pz III by glioma cells was quantified by confocal microscopy. Representative confocal images show porphyrazine III accumulation in cultured astrocytoma (G22), oligodendroglioma (G55), and glioblastoma (G24). Uptake was analyzed during up to 4 h of incubation with Pz III (3 μM). Image (**A**) were acquired at λex 561 nm and λem 600–700 nm using an LSM 710 Axio Observer Z1 DUO NLO laser scanning microscope (Carl Zeiss, Oberkochen, Germany). Image (**B**) was acquired at λex 568 nm and λem 600–750 nm using an LSM 980 with Airyscan 2 laser scanning microscope (Carl Zeiss, Oberkochen, Germany). Scale bars: 20 μm.

Pz III accumulated in glioma cells during a 4 h in vitro incubation, and fluorescence intensity was assessed every hour (Figure 2A). In a low-viscosity medium, this dye exhibits practically no fluorescence, whereas upon entering cells, a significant signal amplification is recorded [42]. It should be noted that a 4 h incubation was sufficient for substantial accumulation of the photosensitizer, which is consistent with previous studies [42,45]. Therefore, this incubation time was chosen for the analysis of photodynamic effects in cells. The dynamics of accumulation in different cultures are presented differently (Figure 2A, Supplementary Figure S2): porphyrazine penetrates astrocytoma G22 cells most rapidly, whereas both oligodendrogliomas exhibit an increase in accumulation intensity after 2 h. The accumulation profile of pz III in glioblastomas reflects a gradual, time-uniform accumulation of the photosensitizer.

Assessment of the intracellular localization of the photosensitizer plays an important role due to the short lifetime and limited diffusion capacity of reactive oxygen species. In other words, the photodynamic efficacy of a photoagent depends on its localization within cellular organelles, as the site of photosensitizer localization becomes the primary target. It has been shown that one of the prerequisites for immunogenic cell death is the production of reactive oxygen species induced by endoplasmic reticulum stress [40]. Furthermore, accumulation of the agent in lysosomes and the Golgi apparatus can lead to immunogenicity of tumor cell death [17].

**Figure 3:**
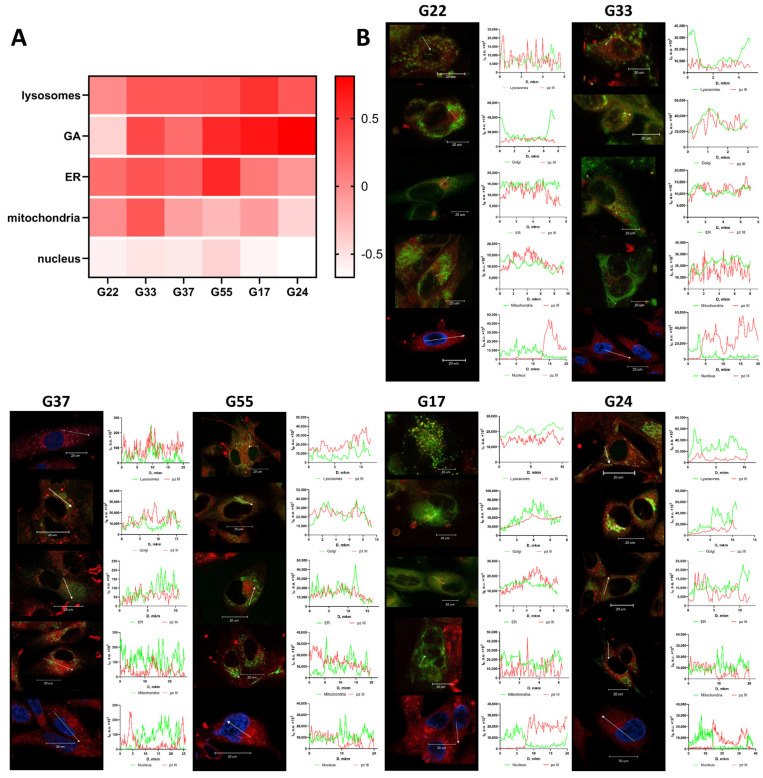
**Study of porphyrazine III distribution in astrocytoma, oligodendroglioma, and glioblastoma cells.** (**A**) The results of Pearson correlation analysis of pz III colocalization with specific trackers of cellular compartments. In G22 astrocytoma cultures, porphyrazine accumulates in the ER; in G33, it is redistributed among the GA, ER, mitochondria, and lysosomes. Pz III accumulates in G37 oligodendroglioma predominantly in lysosomes and the ER, whereas in G55, it accumulates in the ER and Golgi. In glioblastomas (G17, G24), porphyrazine accumulated in the GA and lysosomes. The Pearson correlation coefficient was calculated to assess the colocalization of the photosensitizer and cellular compartment trackers based on pixel fluorescence intensities in confocal microscopy images using a standard two-channel signal correlation algorithm. Colocalization of the photosensitizer and cellular compartment markers was considered sufficient when the Pearson correlation coefficient was >0.3. (**B**) Colocalization was assessed by confocal microscopy after 4 h of photosensitizer pz III (3 μM) accumulation. Fluorescence signal profiles along the lines are indicated by the white arrow on the images with superimposed fluorescence channels. D: distance along the specified segment. Ifl: fluorescence intensity. The following dyes were used: LysoTracker Green for lysosomes, BODIPY FL C5-ceramide for the Golgi, ER-Tracker for the endoplasmic reticulum, MitoTracker Green for mitochondria, and Hoechst for the nucleus.

We observed different distribution patterns of pz III in the different cultures (Figure 3). For two glioblastoma cultures, it was shown that pz III accumulates mainly in the Golgi apparatus and, to a lesser extent, in the lysosomes. However, a different pattern was observed for astrocytomas and oligodendrogliomas: both astrocytomas accumulated the photosensitizer in small amounts in the endoplasmic reticulum, while the G33 cell culture also demonstrated equal localization of the photosensitizer in the mitochondria and Golgi apparatus. Oligodendrogliomas were characterized by accumulation of the pz III in the endoplasmic reticulum, Golgi apparatus and lysosomes.

### 3.3. Pz III-Based Photodynamic Therapy Effectively Induces Cell Death in Various Types of Glioma Cells

**Figure 4:**
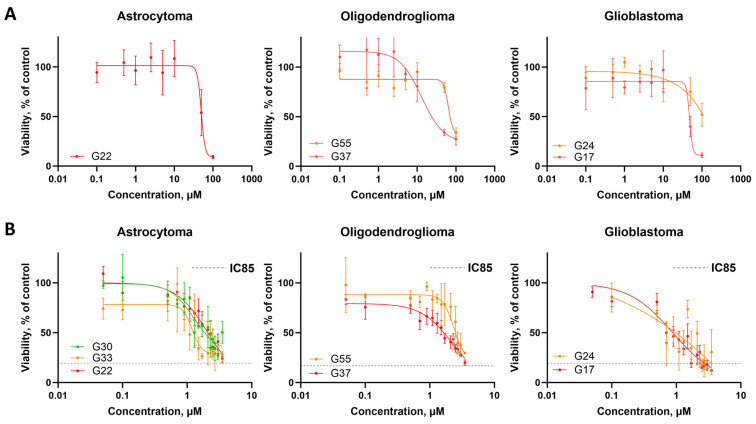
**Pz III-based photodynamic therapy effectively induces cell death in various types of glioma cells.** (**A**) Dark toxicity was assessed in glioma cells after addition of the photosensitizer in serum-free medium for 24 h. Logarithmic dose–response curve showing cell viability (%) after 24 h incubation with photosensitizer (0.1–100 μM) in serum-free medium. Data are means ± SEM (n ≥ 6 per group) for each culture. (**B**) Logarithmic dose–response curve showing cell viability of different glioma cultures 24 h after irradiation. Cell death was induced by incubating the cells with 0.05–3 µM photosensitizer in serum-free medium for 4 h and then exposing them to a light dose of 20 J/cm^2^. IC_85_ for G22 was 4.549 μM [2.064; 7.106], IC_85_ for G33 was 1.595 μM [1.177; 4.647], IC_85_ for G30 was 0.6118 μM [0.2761; 1.034], IC_85_ for G37 was 4.808 μM [2.447; 6.64], IC_85_ for G55 was 1.678 μM [1.230; 2.126], IC_85_ for G17 was 2.820 μM [1.124; 3.311], IC_85_ for G24 was 0.6943 μM [0.5625; 0.9636]. The values were calculated with 95% confidence intervals (three individual experiments with 3 replicates in each).

The dark toxicity of pz III was tested over 24 h without irradiation. A viability assay based on 3-(4,5-dimethylthiazol-2-yl)-2,5-diphenyltetrazolium bromide showed that cell survival was >90% up to 10 μM in the dark (Figure 4A). At concentrations above 10 μM, porphyrazine induced significant dark toxicity.

The cytotoxicity of the photosensitizer was assessed by the MTT assay 24 h after PDT at a dose of 20 J/cm^2^. The IC_85_ values were calculated for each glioma cell culture. IC85—concentration causing 85% cell death or inhibition of a vital process. Toxicity curves for different glioma subtypes allow characterization of resistance to the toxic effect of PDT (Figure 4B). The toxicity threshold zone differs among subtypes: the viability of glioblastoma cells decreases at concentrations exceeding 0.1 μM, whereas for astrocytoma and oligodendroglioma cultures, a decrease in viability was characteristic at concentrations exceeding 0.9 μM. Despite the general shape of the curves, the cell cultures demonstrate different sensitivities to PDT, particularly in the intermediate concentration range: 0.9–2.1 μM. The most resistant cultures at intermediate doses are oligodendrogliomas, whereas glioblastomas exhibit maximal cell death. This may indicate differences in cellular metabolism and redox homeostasis, including previously described antioxidant adaptations in IDH-mutant gliomas, a phenomenon known as the “Warburg shift”—a persistent increase in reactive oxygen species levels due to electron transport chain dysfunction. This lowers the threshold for ROS-mediated peroxidation cascades upon photodynamic therapy activation [47,48].

### 3.4. PzIII-PDT Induces Regulated Cancer Cell Death

Induction of immunogenic cell death is a highly desirable event during photodynamic therapy. From both conceptual and practical standpoints, it is important to understand the features of ICD and to be able to distinguish this process from non-immunogenic types of cell death.

One of the key prerequisites for ICD induction is cytotoxic stress that results in regulated cell death. Critically, cells must activate multiple stress response pathways (which ultimately fail) before undergoing death [49]. In other words, the commitment to die is not an instantaneous cellular decision but rather a process that unfolds over time in response to irreversible damage, such as non-repairable post-translational modifications of peptides.

Annexin V staining is employed to monitor early stages of RCD. This technique relies on the ability of Annexin V to bind phosphatidylserine in a calcium-dependent manner. Under physiological conditions, phosphatidylserine resides in the inner leaflet of the plasma membrane. However, at the early stages of cell death, it aberrantly translocates to the outer leaflet, thereby becoming accessible for detection.

To successfully induce immunogenic cell death, it is critical to achieve a certain level of cytotoxicity. According to the protocol [34], cell death should be induced within the IC75–85 range [50,51]. To characterize the induced cell death, we performed FACS analysis using Annexin-V FITC and Sytox Blue staining. The Annexin-V^−^/Sytox Blue^−^ population reflects viable cells. The Annexin-V^+^/Sytox Blue^−^ population is characterized by phosphatidylserine exposure at the cell surface with preserved integrity of the plasma membrane. The cells in this population are at the early cell death stage. The Annexin-V^+^/Sytox Blue^+^ population reflects dying cells in the later stages. The Annexin-V^−^/Sytox Blue^+^ population is a very minor population representing cells that undergo cell death via the necrotic pathway. The Annexin-V/Sytox Blue staining can detect only the stage of cell death but not the cell death type [42,52,53,54].

**Figure 5:**
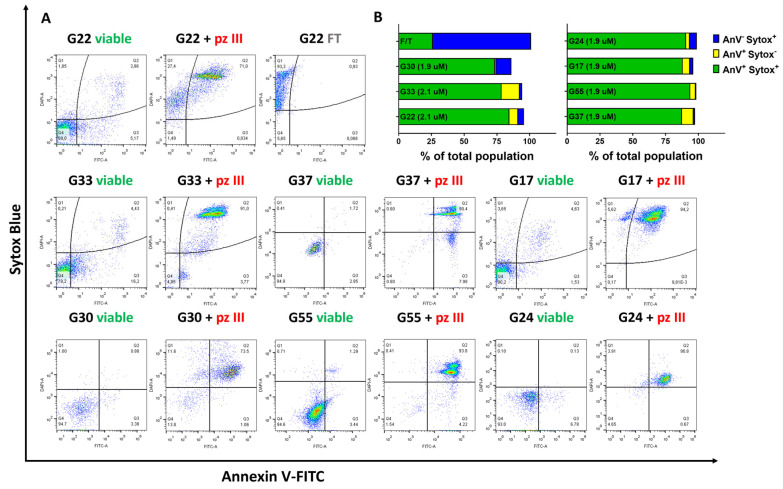
**Primary glioma cells undergo regulated cell death during photodynamic treatment using porphyrazine.** (**A**) All glioma cells die via regulated cell death, as evidenced by the detection of phosphatidylserine on dying cells. The Annexin-V^−^/SYTOX Blue^−^ population reflects viable cells. The Annexin-V^+^/SYTOX Blue^−^ population is characterized by phosphatidylserine exposure at the cell surface with preserved integrity of the plasma membrane. The cells in this population are at the early cell death stage. The Annexin-V^+^/SYTOX Blue^+^ population reflects dying cells in the later stages. The Annexin-V^−^/SYTOX Blue^+^ population is a very minor population representing cells that undergo cell death via necrosis. Dying/dead cells were harvested from the culture plastic 24 h after photoinduction. Cells were washed in Annexin V binding buffer and stained with 0.5 μM SYTOX Blue Nucleic Acid Stain (Molecular Probes) and FITC Annexin V (Invitrogen). The gating strategy was identical across all cultures, taking into account the autofluorescence of live cells and relative to necrotic control cells (freeze-thawed, FT). (**B**) Cell death analysis was performed 24 h after PDT irradiation. Population distribution data for each glioma subtype are presented as the percentage of cells relative to the total cell population, n ≥ 3.

To achieve RCD, the optimal photosensitizer concentration was predetermined for each cell culture (Figure 5). Astrocytomas G22 and G33 were treated with a concentration of 2.1 μM, whereas G30 was treated with 1.9 μM. Notably, the population of necrotically dying cells in G30 was larger than that in the other astrocytomas. Oligodendrogliomas G37 and G55 were induced using 1.9 μM pz III. Both glioblastomas underwent PDT induction with porphyrazine at 1.9 μM. These optimized conditions resulted in the death of 85% or more of the cells, with the predominant fraction undergoing regulated cell death, and the majority of cells being at the late stage of death.

### 3.5. Effect of Different Inhibitors on Cell Death of Glioma Cells Induced by pz III-PDT

Understanding the type of cell death (apoptosis, necroptosis, ferroptosis) induced by photodynamic therapy in the context of ICD analysis is essential for assessing the quality and consequences of the immune response that will be triggered upon tumor cell death. To identify the cell death modality induced by porphyrazine in distinct glioma subtypes, we employed specific cell death inhibitors: z-VAD-fmk (a pan-caspase inhibitor) to block apoptosis, Necrostatin-1s (a RIPK1 inhibitor) to block necroptosis, and Ferrostatin-1 (an inhibitor of reactive oxygen species) to block ferroptosis. It is known that the type of cell death induced by photosensitizers may depend on the photosensitizer, its concentration, and the light dose. In previous work [42], it was shown that pz III-PDT leads to cell death along the pathway of ferroptosis and apoptosis in the case of the murine glioma GL261 cell culture, or along the path of necroptosis and apoptosis in the case of the murine fibrosarcoma MCA205 cell culture.

**Figure 6:**
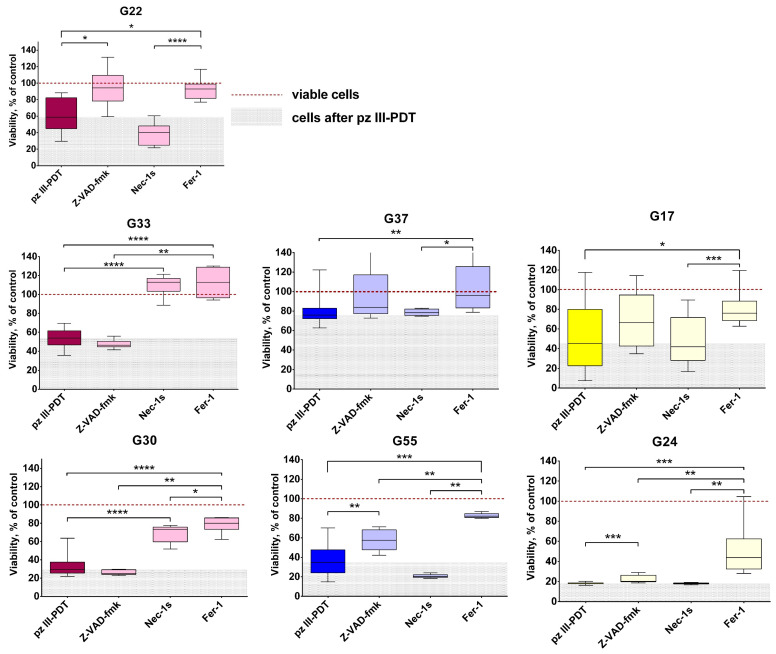
**Effect of different inhibitors on the death of glioma cells induced by pz III-PDT.** The following inhibitors were used to block cell death induced by pz III-PDT: 50 μM Z-VAD-fmk for apoptosis detection, 20 μM Necrostatin-1 s (Nec-1s) for necroptosis detection and 1 μM Ferrostain-1 (Fer-1) or for ferroptosis detection. In this procedure, the cells were pre-incubated with pz III in the presence of the respective cell death blocker in serum-free medium for 4 h. The medium was then replaced by a photosensitizer-free medium, followed by irradiation at 20 J/cm^2^ using an LED light source (615–635 nm). The respective inhibitor was added again before irradiation. 14 h after irradiation, MTT assays were performed. Cell death in G22 astrocytoma cells was significantly blocked by Z-VAD-fmk and Fer-1. In contrast, cell death in G33 and G30 astrocytomas was inhibited by either Z-VAD-fmk and Nec-1 s. In G37 oligodendroglioma cells, cell death was markedly suppressed by Fer-1, whereas in G55 cells, it was inhibited by both Z-VAD-fmk and Fer-1. Fer-1 provided strong inhibition against cell death in G17 glioblastoma cells. In contrast, cell death in G24 cells was effectively inhibited by treatment with either Z-VAD-fmk or Fer-1. Dotted line represents untreated control (no photosensitizer or inhibitor; set as 100% viable cells). The values are the means ± SEM from n  ≥ 12. The Mann–Whitney test was used to calculate statistical significance, * *p*  <  0.05, ** *p* < 0.01, *** *p* < 0.001, **** *p* < 0.0001.

At high concentrations or high light doses, photosensitizers may cause an immediate, uncontrolled cell death called accidental necrosis. Therefore, we chose treatment conditions corresponding to the IC85. After 14 h of pz III-PDT, the effect of apoptosis and ferroptosis inhibitors was evident for G22, G55, and G24 cell cultures. The pan-caspase inhibitor z-VAD-fmk and necrostatin—1s significantly inhibited the death of G33 and G30 cells induced by pz III (Figure 6). These data indicate that pzIII-PDT induces a mixed type of cell death with major ferroptotic components and minor necroptotic or apoptotic components. Indeed, it has been reported that PDT can induce mixed forms of cell death. Importantly, the cell death induced by pz III on G37 culture was inhibited only by Ferrostatin-1, showing that the cells died purely by ferroptosis.

### 3.6. Contribution of Different Types of ROS to Photodynamic Cell Death of Glioma

We investigated whether the type of ROS generated during PDT might depend on the photosensitizer’s subcellular localization, thereby leading to distinct cell death modalities. Given the short lifetime and limited radius of action of reactive oxygen species (especially singlet oxygen), primary damage occurs exactly at the site where the photosensitizer resides at the moment of irradiation. As different organelles possess unique biochemical features and susceptibilities, damage to them activates various cell death signaling pathways.

We investigated the dynamics of ROS production upon photoinduction and identified the predominant ROS using specific probes. The following selective scavengers were employed: NaN_3_ for singlet oxygen (^1^O_2_), D-Mannitol for hydroxyl radical (^●^OH), and Tiron for superoxide (O_2_^●−^) [55].

**Figure 7:**
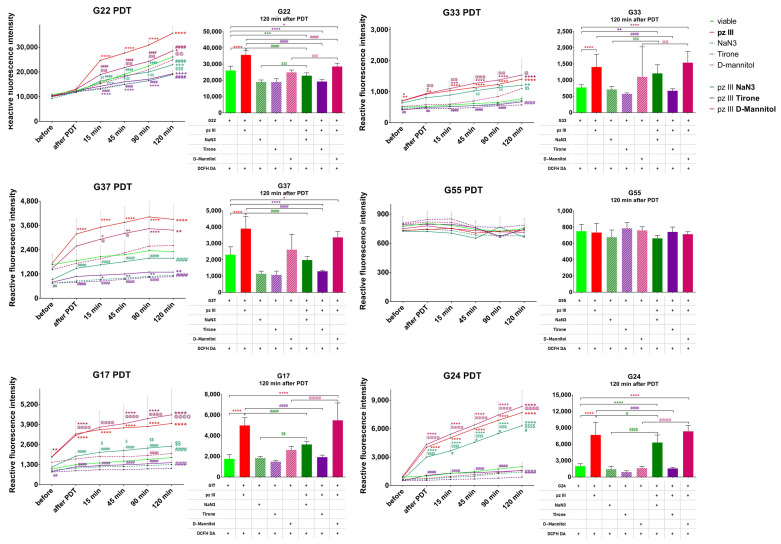
**Determination of the contribution of various ROS forms to photoinduced oxidative stress in glioma cells using selective inhibitors and the DCFH-DA probe.** Cells were incubated for 20 h with selective ROS inhibitors at a concentration of 10 mM: NaN_3_ for singlet oxygen (^1^O_2_), D-mannitol for hydroxyl radical (•OH), and Tiron for superoxide (O_2_•^−^). Then, cells were incubated in serum-free medium simultaneously with pz III and the inhibitors. After the loading step, DCFH-DA staining was performed (10 μM, 30 min) in PBS. Following incubation, the medium was replaced with PBS and fluorescence was recorded (λ_ex_ 488 nm, λ_em_ 525 nm). Irradiation was carried out at a dose of 2 J/cm^2^ (1 min 40 s). Fluorescence was measured immediately after PDT and over a period of 120 min post-PDT. Mean values ± SD are presented, n ≥ 6. The analysis was performed using two-way ANOVA with Tukey’s multiple comparisons test. *—difference from the viable group (cells not subjected to PDT treatment; basal ROS levels in the culture were determined using DCFH-DA). #—difference from the pz III group (cells subjected to pz III-PDT; DCFH-DA detects the entire pool of ROS produced as a result of the treatment). $, &, and @—differences from the NaN_3_, Tiron, and D-mannitol groups, respectively. *, #, $, &, @—*p*  <  0.05, **, ##, $$, &&, @@—*p* < 0.01, ***, ###, $$$, &&&, @@@—*p* < 0.001, ****, ####, $$$$, &&&&, @@@@—*p* < 0.0001.

To confirm the ability of the tested conjugates to induce a photodynamic reaction in living cells, intracellular ROS generation was monitored using the 2′,7′-dichlorodihydrofluorescein diacetate (DCFH-DA) probe. DCFH-DA freely penetrates into cells, where it is hydrolyzed by esterases to 2′,7′-dichlorodihydrofluorescein (DCFH), which is subsequently oxidized by intracellular ROS to fluorescent 2′,7′- dichlorofluorescein (DCF) (Figure 7) [56]. The fluorescence of cells incubated with DCFH-DA without light irradiation (“before” group) in glioma G22, G24, and G37 insignificantly exceeded the control, indicating a low level of basal ROS production in the cells. In contrast, photosensitizer-loaded G17, G33, and G55 cell cultures already differed from unloaded cells prior to irradiation. This indicates that even the dark toxicity of the photosensitizer is sufficient to generate ROS, a trend that continues to be observed over time (Supplementary Figure S3). Light irradiation of the cells produced a consistent increase in fluorescence intensity over 120 min following brief irradiation (1 min 40 s) in all cultures except G55 (Figure 7). Hence, photoinduction led to a sustained rise in fluorescence intensity for 2 h post-irradiation. A marked difference in DCF fluorescence relative to control values (viable vs. pz III) was observed immediately post-irradiation in G17, G24, and G37 cultures, whereas pronounced ROS release was evident at 15 min in G22 and G33 cultures. The intense fluorescence from viable cells reflects ongoing active oxidative processes.

At the same time, the key type of ROS induced by cyano-aryl-porphyrazine in all cultures was the superoxide anion (pz III vs. Tiron—pz III). In G17, G22, G33, and G37 cultures, singlet oxygen also made a significant contribution (pz III vs. NaN_3_—pz III). However, the formation of hydroxyl radical, which was scavenged by D-mannitol (pz III vs. mannitol—pz III), was detected in only one culture—G22. Detailed statistics on the temporal dynamics are presented in Supplementary Table S1.

### 3.7. Changes in Mitochondrial Status During Cell Death

Even though the localized in mitochondria, mitochondria can themselves be regarded as a key target of damage. Given the short diffusion distance of reactive oxygen species, primary oxidative events are confined to this compartment, which may initiate mitochondrial apoptosis cascades via ATP synthesis arrest and disruption of energy homeostasis.

**Figure 8:**
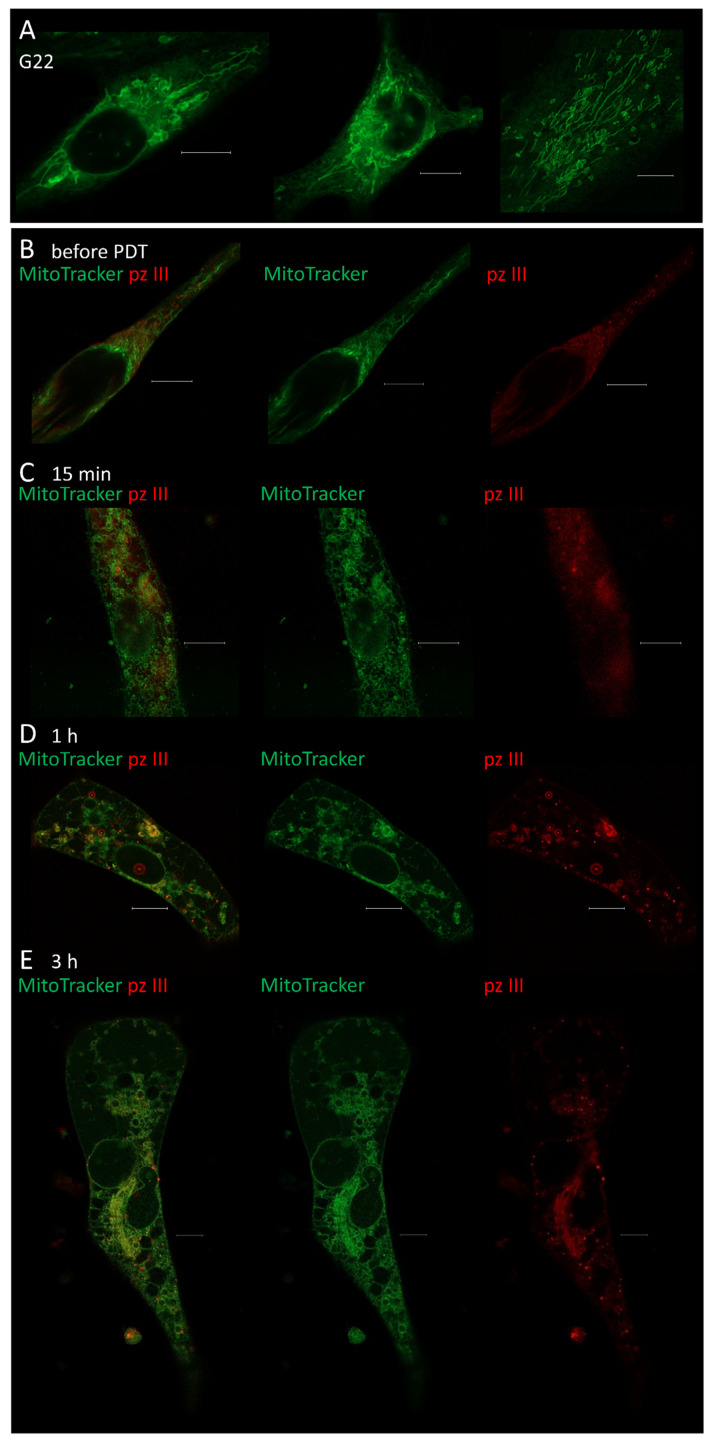
**Time-dependent disruption of the mitochondrial network in G22 human astrocytoma cells following pz III-mediated photodynamic therapy**. Representative confocal images (100×) of live G22 cells stained with MitoTracker Green (λex = 488 nm, λem = 495–550 nm; green) to visualize mitochondrial morphology, and with pz III (λex = 631 nm, λem = 650–700 nm; red). Scale bars: 10 μm. (**A**) Untreated control cells display a highly organized, interconnected tubular mitochondrial network with elongated morphology. (**B**) After 4 h of dark incubation with pz III (no irradiation), the mitochondrial network remains intact and morphologically indistinguishable from controls, consistent with predominant pz III localization to the endoplasmic reticulum rather than mitochondria at this stage. Following PDT (irradiation after the 4 h incubation), time-dependent mitochondrial disruption is observed: (**C**) at 15 min post-PDT, the tubular network rapidly fragments into enlarged, globular perinuclear clusters; (**D**) by 1 h post-PDT, fragmentation intensifies with further aggregation; (**E**) by 3 h post-PDT, mitochondria exhibit severe dysmorphology and are frequently associated with autophagic vesicles. Partial colocalization of pz III (red) with mitochondria (green) is observed only after irradiation (**C**–**E**), appearing as yellow regions in merged channels.

Primary glioma G22 cells in untreated controls exhibited a highly organized mitochondrial network of elongated, spindle-shaped tubules, as visualized by MitoTracker Green staining (Figure 8A). Incubation with pz III for 4 h induced no morphological alterations, confirming its predominant localization to the endoplasmic reticulum and lysosomes rather than mitochondria (Figure 8B). At 15 min after PDT (Figure 8C), the tubular network underwent rapid fragmentation into enlarged, globular clusters, with partial colocalization of pz III fluorescence and mitochondrial structures observed at discrete subcellular sites. By 1 h after PDT (Figure 8D), mitochondrial fragmentation further intensified. By 3 h (Figure 8E), mitochondria retained marked dysmorphology and were frequently found within autophagic vesicles, consistent with ongoing organelle clearance and irreversible mitochondrial dysfunction, ultimately leading to cell death.

Loss of mitochondrial membrane potential is considered a key indicator of cellular function, and measuring its changes is crucial for monitoring the process of regulated cell death. The JC-1 dye was used to assess the membrane potential ΔΨm. Under normal mitochondrial microenvironment conditions with standard membrane potential, JC-1 accumulates in negatively charged mitochondria, forming red fluorescent aggregates. In contrast, in cells undergoing apoptosis, JC-1 penetrates mitochondria to a lesser extent due to increased membrane permeability and loss of the electrochemical gradient, thus remaining in the monomeric form with green fluorescence.

**Figure 9:**
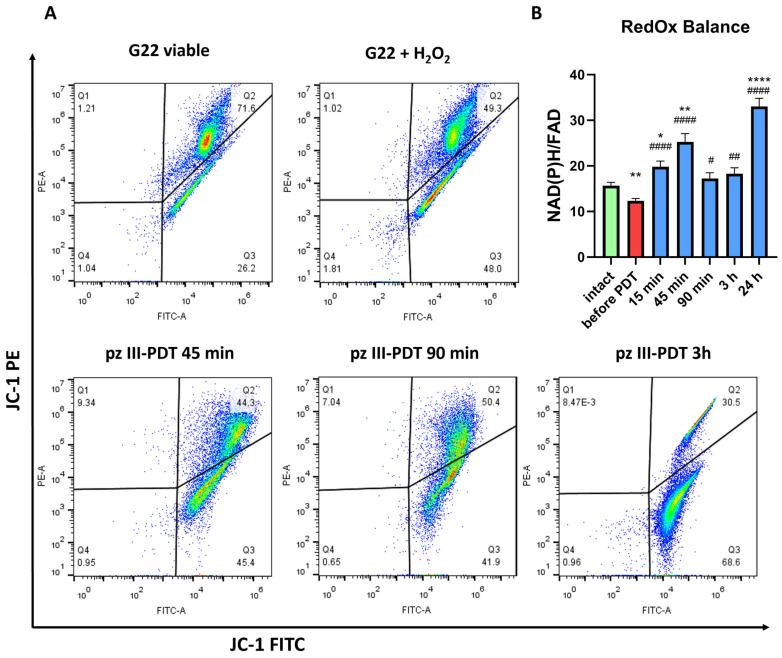
**PDT induces mitochondrial dysfunction in primary glioma cultures.** (**A**) Flow cytometry dot plots showing mitochondrial membrane potential assessed with JC-1 dye: viable G22 cells (intact), H_2_O_2_-treated G22 cells (depolarization control), and cells at 45 min, 90 min, and 3 h after pz III-PDT. (**B**) Graph of the redox balance (NAD(P)H/FAD ratio) based on confocal microscopy autofluorescence intensities. Groups: intact (untreated glioma cells), before PDT (4 h incubation with photosensitizer without irradiation), and cells at 45 min, 90 min, 3 h, and 24 h after pz III-PDT. Values are means  ±  SEM (n  ≥  30). The Mann–Whitney test was used to calculate statistical significance. Significance symbols: *, #—*p* < 0.05; **, ##—*p* < 0.01; ****, ####—*p* < 0.0001. *—vs. “intact”; #—vs. “before PDT”.

Flow cytometric analysis revealed that by 45 min following PDT, 45.4% of G22 culture cells had already lost J-aggregates, indicating a reduction in mitochondrial potential (Figure 9A). By 3 h post-exposure, the fraction of depolarized cells reached 68.6%, reflecting a marked and nearly irreversible collapse of the proton gradient associated with the opening of the mitochondrial permeability transition pore (mPTP).

Additionally, the dynamics of the redox balance were studied based on the spontaneous autofluorescence of NAD(P)H and FAD (Figure 9B). In untreated cells, the NAD(P)H/FAD ratio averaged 15.6 ± 0.7. After a 4 h incubation with porphyrazine III in the absence of light irradiation, a certain decrease in this parameter was observed, down to 12.3 ± 0.5, which may indicate an initial stress on the respiratory chain even under ‘dark’ conditions. After PDT, within 15 min, the ratio increased to 19.83 ± 1.2, peaked at 45 min, reaching 25.3 ± 1.8, and then decreased by 1.5 h to 17.3 ± 1.2 and by 3 h to 18.3 ± 1.3. The highest values of the redox balance were recorded at 24 h, when the NAD(P)H/FAD ratio reached 33.0 ± 1.8 in completely dead cells. The pronounced hyperreduction may indicate inhibition of respiratory complexes I/III, activation of reverse electron flow through NADH dehydrogenase, and blockade of ATP synthase, leading to a significant energy deficit.

During photodynamically induced cell death, prolonged mitochondrial responses to photoirradiation are observed: a rapid ‘acute’ phase within the first 45 min, characterized by rapid depolarization of the mitochondrial membrane with pronounced hyperproduction of NAD(P)H, followed by a prolonged partial recovery of the NAD(P)H/FAD ratio against the background of sustained mitochondrial potential dysfunction.

## 4. Discussion

Induction of immunogenic cell death in the context of cancer immunotherapy represents a highly promising strategy [49,57,58]. Educating the immune system to combat tumors may serve as an effective approach for targeting cancer cells, including highly malignant gliomas [32]. In this context, photodynamic therapy offers distinct advantages as an inducer of ICD.

Tetra(aryl)tetracyanoporphyrazines belong to the class of porphyrazines and share a high degree of structural similarity with the phthalocyanine group [59,60,61]. Phthalocyanines are a widely studied group that have proven to be effective photodynamic agents [62,63,64], as well as inducers of ICD [5,65]. The high solubility of these compounds, along with their rapid accumulation in tumor cells, makes them convenient for use under controlled laboratory conditions. Alongside ICD inducers, porphyrazine III triggers the release of DAMPs and also primes tumor antigens for uptake by dendritic cells, which is accompanied by their maturation and readiness for antigen presentation [19,42].

The main challenge of this work was to transition from simplified mouse tumor models to primary patient tissue obtained during surgical intervention. The complexity lies in interpatient heterogeneity, the unpredictability of tumor growth in vitro, as well as the need to select individual conditions for cell death induction for each individual culture. Nevertheless, the study of the toxic properties of the porphyrazine in cell cultures revealed that a concentration range of 1.9–2.1 µM induces 85% cell death in all cultures. However, cell death proceeds at different rates and intensities. This is likely explained by the primary target of the photosensitizer. Porphyrazine III effectively accumulates in the Golgi apparatus and the ER in GL261 mouse glioma cells [19] and in the ER and lysosomes in MCA205 cells [42]. In the present study, we determined that the photosensitizer accumulates differently in different patient-derived gliomas. This may critically depend on the physiological activity of the cells, their metabolic status, and susceptibility to stress. Empirically, we observed that all gliomas included in the study exhibited different subculturing rates, distinct morphologies, varying mitotic activities, and different growth patterns on adhesive plastic. We observed the distribution of porphyrazine III to the Golgi apparatus, lysosomes, mitochondria, and ER across different cultures. It is known that the accumulation of photosensitizers in the Golgi apparatus [17], lysosomes [66], mitochondria [67] and ER [40,68] leads to ICD in various studies using mouse tumor models. A possible scenario also involves the redistribution of the photodye within cells among organelles [69,70,71,72], although this requires detailed time-course studies.

Generation of ROS in different compartments seemingly triggers specific damage cascades. Based on our data and previously published findings, it can be hypothesized that accumulation of pz III in the Golgi apparatus induces stress, which, as shown in [73,74], activates the NLRP3 inflammasome and caspase-1. The latter likely contributes to apoptosis via cytochrome c release. Damage to lysosomes appears to disrupt membrane integrity, releasing cathepsins and iron ions, which trigger the Fenton reaction and ferroptosis. Moreover, cathepsins B and D may activate Bid, thereby stimulating mitochondrial apoptosis. It is possible that the combined induction of apoptosis and ferroptosis is explained by synergistic damage to lysosomes and the Golgi apparatus [75]. In the endoplasmic reticulum, local ROS likely activate the PERK-eIF2α-ATF4-CHOP pathway. This may increase PUMA expression, triggering p53-independent apoptosis and ferroptosis through glutathione depletion [75,76]. Upon distribution of pz III to mitochondria, one might expect disruption of the respiratory chain and GPX4, leading to ferroptosis, as well as apoptosis (Bax/Bak) and necroptosis (RIPK1) [77].

Furthermore, the broad distribution of porphyrazine III across different organelles may indicate the activation of mixed cell death pathways with distinct dynamics [78]. Such multicentric photodynamic therapy is likely more effective, as it induces cellular ‘collapse’ on multiple fronts, thereby preventing adaptation. Consequently, the transition from mouse models to primary patient tissue has revealed both new challenges for investigation (variability in photosensitizer localization) and a key advantage—the presence of a universal «concentration window».

An important stage of this work is the assessment of the ability to trigger regulated forms of cell death. We employed a classic cytofluorometric protocol to evaluate the redistribution of phosphatidylserine to the cell surface in response to induced stress, which serves as a marker of regulated, energy-dependent cell death [79]. We have demonstrated that the selected photoinduction conditions lead to RCD with high reproducibility. We consider this step critical, as it is necessary in all experimental studies for further investigation of DAMP release and the specific induction of antigen presentation. The ability to consistently undergo cell death following photoinduction under defined conditions is an important finding for subsequent translational implementation of the method. This fact has also been repeatedly confirmed in mouse glioma models [80,81,82].

In addition to RCD, our focus was directed toward the type of cell death in gliomas following photoinduction. Previously, while studying the types of cell death induced in a glioma cell line, we observed apoptosis with a contribution from ferroptotic cell death (data not published). In MCA205 cells, pz III induced mixed cell death involving both apoptotic and necroptotic pathways [42]. In primary cultures, we observe ferroptotic cell death with an admixture of apoptosis and necroptosis across different cultures. The induction of ferroptosis following PDT affects intracellular redox homeostasis, thereby increasing the susceptibility of cancer cells to oxidative stress. Furthermore, PDT also stimulates ferroptosis by triggering lipid peroxidation [83,84,85]. PDT and ferroptosis exert a mutually reinforcing effect, demonstrating high antitumor efficacy, which makes them a promising strategy for cancer treatment [86]. Moreover, the release of DAMPs from tumor cells undergoing ferroptosis can activate antigen-presenting cells, thereby modulating the adaptive immune response [30,82,87,88,89,90]. Mixed cell death pathways also exhibit similar activity [91]. It is well established that necroptosis induced by PDT is associated with the release of damage-associated molecular patterns, thereby inducing immunogenic cell death [92,93,94,95,96]. Thus, the ferroptotic cell death we have observed, occurring with contributions from apoptosis and necroptosis, may potentially induce immunogenic cell death, which is critically important for the translational application of this concept.

We anticipated that cell death might depend on the subcellular localization of the photosensitizer; however, no correlation was observed. Apparently, stress originating from different organelles triggers distinct biochemical cascades leading to programmed cell death via different pathways. It is also known that the same photosensitizer can induce different types of cell death if its localization changes. A classic example is zinc(II) phthalocyanine (ZnPc). Following a short incubation, it accumulated in the Golgi apparatus and induced necrosis. Following a prolonged incubation, it reached the mitochondria, and cell death shifted toward apoptosis [97].

Alongside the localization of the photosensitizer, the type of ROS generated plays an important role. It is known that the most potent type of ROS—singlet oxygen (^1^O_2_)—has an extremely short lifetime (less than 0.04 microseconds) and a small diffusion radius (less than 0.02 μm). It is unable to diffuse far from its site of generation [98]. In essence, the photosensitizer affects only the organelle in which it resides, acting like a point detonator [99]. We investigated which type of ROS predominates upon induction by porphyrazine III in glioma cells, and the most significant contribution is made by the superoxide anion. We propose that the superoxide anion, as a product of the type I photodynamic reaction, triggers a chain reaction capable of gradually accumulating damage and switching the mode of cell death [100]. However, we do not rule out the occurrence of secondary ROS production [71]. Superoxide dismutase converts the superoxide anion into hydrogen peroxide [101]. H_2_O_2_ freely crosses membranes and induces sequential, slow stress responses in cellular structures. It is important for us to achieve gradual cell death, as this is one of the conditions for immunogenicity of cell death. The mechanism of adaptive immune response activation by various reactive oxygen species triggered by photodynamic therapy remains insufficiently studied [102,103], and we see a wide field for research in this area. The mechanism of ferroptosis was described by the group of Patrizia Agostinis upon ROS generation at the endoplasmic reticulum–mitochondria contact sites (EMCSs) [104]. It is reported that phospholipid peroxidation primarily occurs in the endoplasmic reticulum but can spread to other compartments through the formation of membrane contacts with them. This process subsequently extends to the mitochondria, leading to the generation of mitochondrial ROS (secondary ROS production). The ensuing accumulation of cellular lipid peroxides, exacerbated by mitochondrial damage, ultimately results in ferroptosis.

We studied changes in mitochondrial potential following photodynamic treatment. The pronounced and rapid drop in mitochondrial membrane potential, observed as early as 45–90 min after PDT, is typical of the execution of the mitochondrial pathway of apoptosis. It is associated with the opening of the mPTP pore, the release of cytochrome c, and the activation of the caspase cascade network [105,106]. Following this logic, membrane depolarization is regarded as a key step in the ‘mitochondrial switch’ from an attempt at recovery to irreversible programmed cell death. Apparently, superoxide anions generated during photoinduction act as the primary damaging agent. They not only act directly but also activate secondary ROS generation in mitochondria and trigger the disruption of the inner mitochondrial membrane. We also demonstrate that during cell death, the photosensitizer can redistribute to mitochondria (colocalization with a mitochondrial dye is observed after 1 h by confocal microscopy); however, this is a consequence rather than the primary cause. At the same time, current evidence indicates that mitochondria are also involved in the development of ferroptosis, although its morphological and biochemical profiles differ from those of classical apoptosis [107]. In ferroptosis, changes in mitochondria are also described: a reduction in the size and number of organelles, condensation of the inner membranes, shrinkage or disappearance of cristae, and sometimes a decrease in mitochondrial potential [108,109]. These changes are associated with enhanced iron-dependent lipid peroxidation and depletion of the glutathione/GPX4 antioxidant system, rather than solely with classical caspase-dependent mechanisms [110]. Furthermore, in ferroptosis, the energy metabolism of mitochondria is altered: oxidative phosphorylation and ATP production are increased, while glycolysis is correspondingly reduced [111]. It has been reported that mitochondrial metabolic activity plays a role in generating sufficient lipid ROS to initiate ferroptosis [104,107,109]. However, the mechanism remains poorly understood and requires further experimental validation.

Before drawing final conclusions, it is important to acknowledge certain limitations inherent to this study. Some patients had received treatment prior to the resection of the material (true for G33, G37, G17—material obtained after recurrence), while others had not received any treatment before surgery. We did not account for this factor in the study, but we always kept it in consideration. We observed no differences in the cell death process between treated and untreated tumors. Nevertheless, this fact remains a limitation of our work.

Furthermore, we used a small sample size in this study, which is related to the difficulty of working with primary cultures and the need for individual optimization of experimental procedures with respect to the physiology of the cells. In addition, we performed technical replicates of the experiments, although cultures of the same subtype could have been considered biological replicates. However, while examining the cell death process in these cells, we concluded that the high interpatient heterogeneity within a given subtype dictates an individualized approach.

Although our work includes functional (cell death inhibition by Fer-1), biochemical (superoxide anion analysis as the main type of ROS), and structural (mitochondrial morphology and mitochondrial potential) verification of ferroptosis, we acknowledge that additional evidence of ferroptosis using molecular or biochemical methods could further strengthen our conclusions. This represents a direction for future research.

## 5. Conclusions

On the path toward achieving controlled photoinduction of cell death, we have learned to achieve uniform, regulated, and predictable death of primary glioma cells using porphyrazine III. A striking finding is that cell death proceeds via the ferroptosis pathway in all patient-derived cultures, even though we observe accumulation of the photosensitizer in different organelles in gliomas from different patients. However, in addition to ferroptosis, contributions of apoptosis and necroptosis are observed across different cultures. Since ferroptosis, apoptosis, and necroptosis may possess immunogenic potential, we hypothesize that the induced ferroptosis is also capable of leading to immunogenic cell death. Testing this hypothesis and characterizing the immunogenicity of photoinduced cell death—specifically, the release of DAMPs such as CRT, HMGB1, ATP, and HSP70—will constitute the next stage of our research.

## Figures and Tables

**Table 1:** Clinical Characteristics of Human Gliomas Used to Derive Cell Cultures.

Culture ID	Patient Sex, Age	Diagnosis	IDH Mutation Status (from Clinical Data)
G22	woman, 40	Astrocytoma, Grade III	IDH mutant
G33	man, 37	Astrocytoma, Grade III	IDH mutant
G30	man, 49	Astrocytoma, Grade IV	IDH mutant
G37	woman, 58	Oligodendroglioma, Grade III	IDH mutant
G55	woman, 62	Oligodendroglioma, Grade III	IDH mutant
G17	woman, 46	Glioblastoma, Grade IV	IDH wildtype
G24	man, 64	Glioblastoma, Grade IV	IDH wildtype

**Figure A1:**
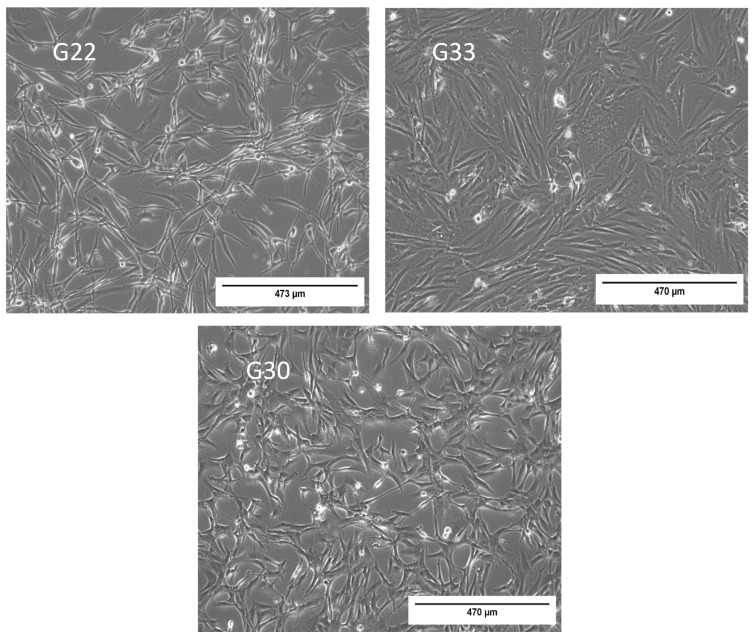
**Morphological characteristics of primary human astrocytoma cell cultures**. Phase-contrast images of primary cultures derived from patient astrocytomas. Cells were imaged at passages 3–4, at least 24 h after subculturing, using the inverted EVOS M5000 Imaging System (Invitrogen, Thermo Fisher Scientific) at 20× magnification. Scale bar: 470 μm.

**Figure A2:**
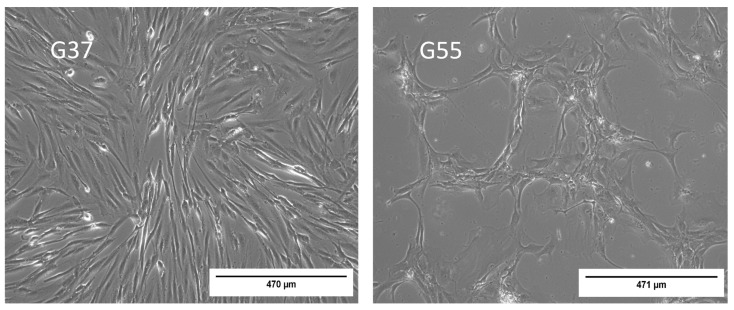
**Morphological characteristics of primary human oligodendroglioma cell cultures**. Phase-contrast images of primary cultures derived from patient oligodendrogliomas. Cells were imaged at passages 3–4, at least 24 h after subculturing, using the inverted EVOS M5000 Imaging System (Invitrogen, Thermo Fisher Scientific) at 20× magnification. Scale bar: 470 μm.

**Figure A3:**
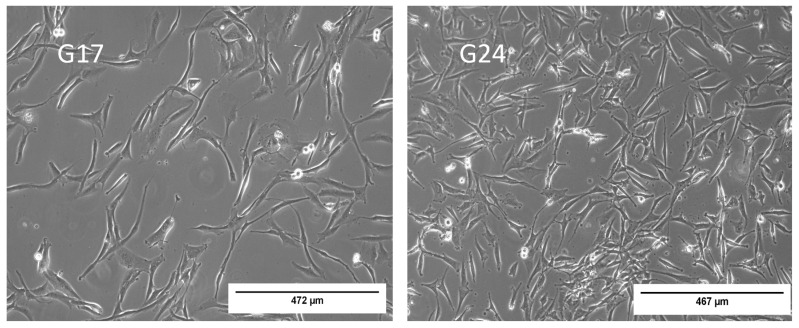
**Morphological characteristics of primary human glioblastoma cell cultures.** Phase-contrast images of primary cultures derived from patient glioblastomas. Cells were imaged at passages 3–4, at least 24 h after subculturing, using the inverted EVOS M5000 Imaging System (Invitrogen, Thermo Fisher Scientific) at 20× magnification. Scale bar: 470 μm.

## Data Availability

The original data presented in the study are openly available in BioStudies at https://www.ebi.ac.uk/biostudies/bioimages/studies/S-BIAD3271?key=9502c10a-04a7-45e1-ba4c-895526846cad (accessed on 21 April 2026).

## Supplementary Materials

The following supporting information can be downloaded at: https://www.mdpi.com/article/10.3390/pharmaceutics18060705/s1, Figure S1: A chemical structure of the synthesized tetracyanotetra(aryl)porphyrazine; Figure S2: Dynamics of pz III accumulation in primary human glioma cell cultures. Representative confocal images of porphyrazine III accumulation in cultured astrocytoma (G33), oligodendroglioma (G55), and glioblastoma (G24). Uptake was analyzed during up to 4 h of incubation with pz III (3 μM). Images were acquired at λex 561 nm and λem 600–700 nm; scale bars: 20 μm; Figure S3: Determination of the contribution of various ROS forms to oxidative stress in pz III-loaded glioma cells using selective inhibitors and the DCFH-DA probe. Cells were incubated for 20 h with selective ROS inhibitors at a concentration of 10 mM: NaN_3_ for singlet oxygen (^1^O_2_), D-mannitol for hydroxyl radical (•OH), and Tiron for superoxide (O_2_•^−^). Then, cells were incubated in serum-free medium simultaneously with pz III and the inhibitors for 4 h. After the loading step, DCFH-DA staining was performed (10 μM, 30 min) in PBS. Following incubation, the medium was replaced with PBS, and fluorescence was recorded (λex 488 nm, λem 525 nm). Fluorescence was measured over a period of 120 min after changing the medium. Mean values ± SD are presented, n ≥ 6. The analysis was performed using two-way ANOVA with Tukey’s multiple comparisons test. *—difference from the viable group (cells not subjected to PDT treatment; basal ROS levels in the culture were determined using DCFH-DA). #—difference from the pz III group (cells subjected to pz III-PDT; DCFH-DA detects the entire pool of ROS produced as a result of the treatment). $, &, and @—differences from the NaN_3_, Tiron, and D-mannitol groups, respectively. *, #, $, &, @—*p*  <  0.05, **, ##, $$, &&, @@ —*p* < 0.01, ***, ###, $$$, &&&, @@@ —*p* < 0.001, ****, ####, $$$$, &&&&, @@@@ —*p* < 0.0001; Table S1: Statistical analysis of differences between groups in the experiment evaluating the type of ROS produced by primary cultures following photodynamic treatment using porphyrazine III (pz III). The analysis was performed using two-way ANOVA with Tukey’s multiple comparisons test.

## Abbreviations

The following abbreviations are used in this manuscript:

APC	Antigen-presenting cell
ANOVA	Analysis of variance
BSA	Bovine serum albumin
CO_2_	Carbon dioxide
CRT	Calreticulin
DAMPs	Damage-associated molecular patterns
DCF	2′,7′ Dichlorofluorescein
DCFH	2′,7′ Dichlorodihydrofluorescein
DCFH DA	2′,7′ Dichlorodihydrofluorescein diacetate
DC	Dendritic cell
DMSO	Dimethyl sulfoxide
ER	Endoplasmic reticulum
FACS	Fluorescence-activated cell sorting
FAD	Flavin adenine dinucleotide
Fer-1	Ferrostatin-1 (inhibitor of ROS and lipid peroxidation)
FITC	Fluorescein isothiocyanate
FBS	Fetal bovine serum
FT	Freeze thawed
GA	Golgi apparatus
GBM	Glioblastoma
GL261	Murine glioma GL261 cell line
HMGB1	High mobility group box 1
Hsp70	Heat shock protein 70
Hsp90	Heat shock protein 90
IC80	Concentration causing 80% inhibition of cell viability
IC85	Concentration causing 85% inhibition of cell viability
ICD	Immunogenic cell death
IDH	Isocitrate dehydrogenase
Ifl	Fluorescence intensity
JC 1	5,5′,6,6′ Tetrachloro 1,1′,3,3′ tetraethylbenzimidazolylcarbocyanine iodide
LED	Light-emitting diode
LSM	Laser scanning microscope
MCA205	Murine fibrosarcoma cell line
mPTP	Mitochondrial permeability transition pore
MTT	3 (4,5 Dimethylthiazol 2 yl) 2,5 diphenyltetrazolium bromide
NAD(P)H	Reduced nicotinamide adenine dinucleotide (phosphate)
Nec 1s	Necrostatin 1s (RIPK1 inhibitor)
O_2_	Molecular oxygen
OH·	Hydroxyl radical
O_2_·^−^	Superoxide anion radical
PBS	Phosphate-buffered saline
PDT	Photodynamic therapy
PS	Photosensitizer
Pz III	Tetra(aryl)tetracyanoporphyrazine with 4-(4-fluorobenzyloxy)phenyl group (photosensitizer prototype)
RCD	Regulated cell death
RIPK1	Receptor-interacting serine/threonine protein kinase 1
ROI	Region of interest
ROS	Reactive oxygen species
SEM	Standard error of the mean
SD	Standard deviation
WHO	World Health Organization
WHO CNS5	Fifth edition of the World Health Organization classification of tumours of the central nervous system
Z VAD fmk	Carbobenzoxy valyl alanyl aspartyl [O methyl] fluoromethylketone
ZnPc	Zinc phthalocyanine
λex	Excitation wavelength
λem	Emission wavelength
ΔΨm	Mitochondrial membrane potential
NaN_3_	Sodium azide
^1^O_2_	Singlet oxygen

## Appendix A

This study used 7 primary human glioma cultures: 3 astrocytomas (G22, G33, and G30), 2 oligodendrogliomas (G37 and G55), and 2 glioblastomas (G17 and G24). A characteristic feature of these cell cultures is the fibroblast-like or spindle-shaped morphology of the cells. The cells of glioblastomas G17 and G24, astrocytomas G30 and G33, and oligodendroglioma G37 exhibit an elongated shape with pointed ends. In the cultures of astrocytoma G22, oligodendroglioma G55, and a subpopulation of G37, polygonal, triangular, or stellate cells with numerous processes predominate. Large flat cells are particularly prominent, and in culture G55, multipolar stellate cells with radially extending processes are distinctly pronounced. Pleomorphism, i.e., the diversity in cell size and shape, is observed in all cultures studied. The range of forms varies from small compact elements to elongated filamentous structures. Loose cell clusters and networks formed by cellular processes are noted, being especially pronounced in cultures G22 and G24.

In astrocytoma cultures, G22, G30, and G33, the cytoplasm is typically abundant or moderately abundant, finely or heterogeneously granular, with distinct perinuclear condensation zones and occasional small vacuoles and round inclusions corresponding to organelles or endocytic vesicles (Figure A1). In oligodendroglioma cells of cultures G37 and G55, the cytoplasm is either abundant or thin at the periphery and denser in the perinuclear region, heterogeneously granular with a fine-grained structure and small vacuoles (Figure A2). In the large cells of culture G37, cytoskeletal elements are clearly distinguishable. The cytoplasm in glioblastoma cultures G17 and G24 is moderately abundant to abundant, heterogeneously granular, with areas of denser granular content and clusters of organelles in the perinuclear region (Figure A3). In some cells, small vacuoles, which may reflect active metabolism, autophagy, or early dystrophic changes.

The nuclei of tumor cells are large, most often oval or slightly irregular in shape, and are located eccentrically. In spindle-shaped cells from cultures G17, G30, G33, and G37, the nuclei are often elongated along the longitudinal axis of the cell. Chromatin exhibits moderate or focal condensation with compacted areas. One to three small nucleoli are observed, indicating high transcriptional and proliferative activity. The nuclear-to-cytoplasmic ratio is high in small elongated cells and moderate in large flattened elements.

The contours of the cells are irregular, with a serrated appearance in places and small cytoplasmic protrusions. Long, thin processes are ubiquitously present, forming intercellular bridges and networks. Peripheral lamellipodial extensions, particularly prominent in cultures G30, G33, G37, and G55, indicate active cytoskeletal remodeling and a high migratory-invasive potential of these gliomas cell cultures.
