# Supplementary material for: Ferroptosis with Contributions from Apoptosis and Necroptosis in Porphyrazine III-Based Photodynamic Therapy of Primary Human Gliomas
Source: Pharmaceutics. 2026 Jun 8;18(6):705. doi: 10.3390/pharmaceutics18060705 (PMC13305198; doi:10.3390/pharmaceutics18060705)
Supplement: Supplementary file 1 [file pharmaceutics-18-00705-s001.zip › pharmaceutics-4289944-supplementary.pdf]

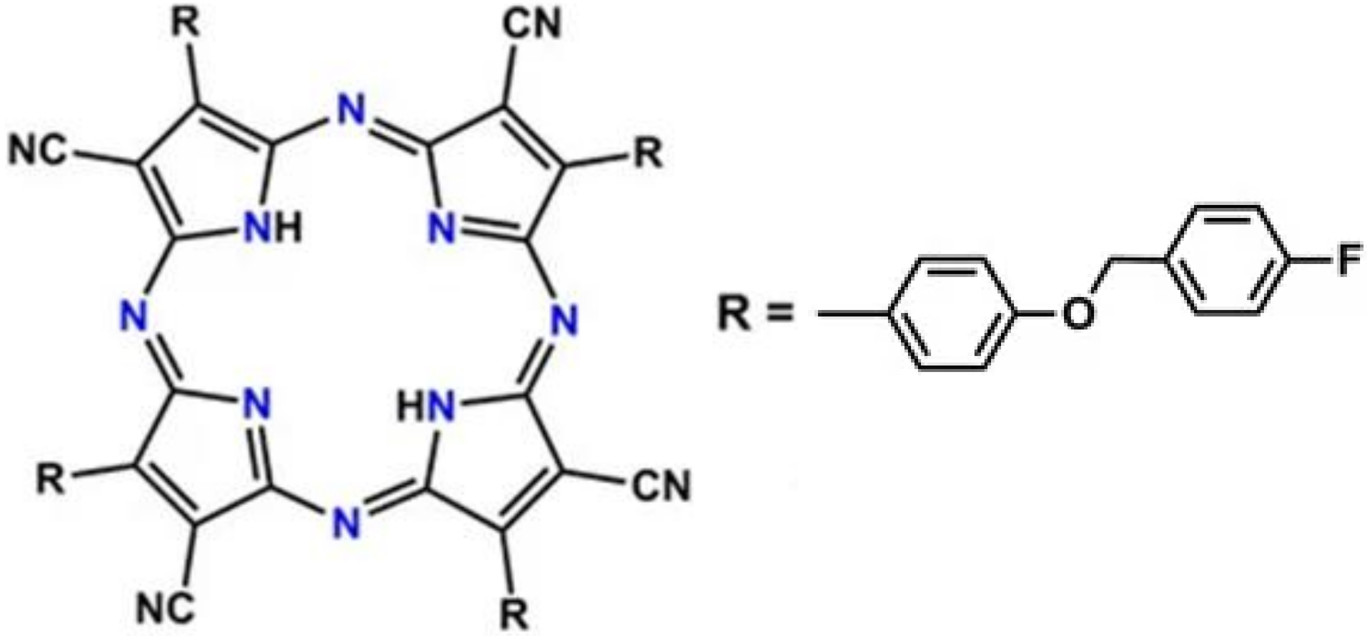

Supplementary figure 1. A chemical structure of the synthesized tetracyanotetra(aryl)porphyrazine

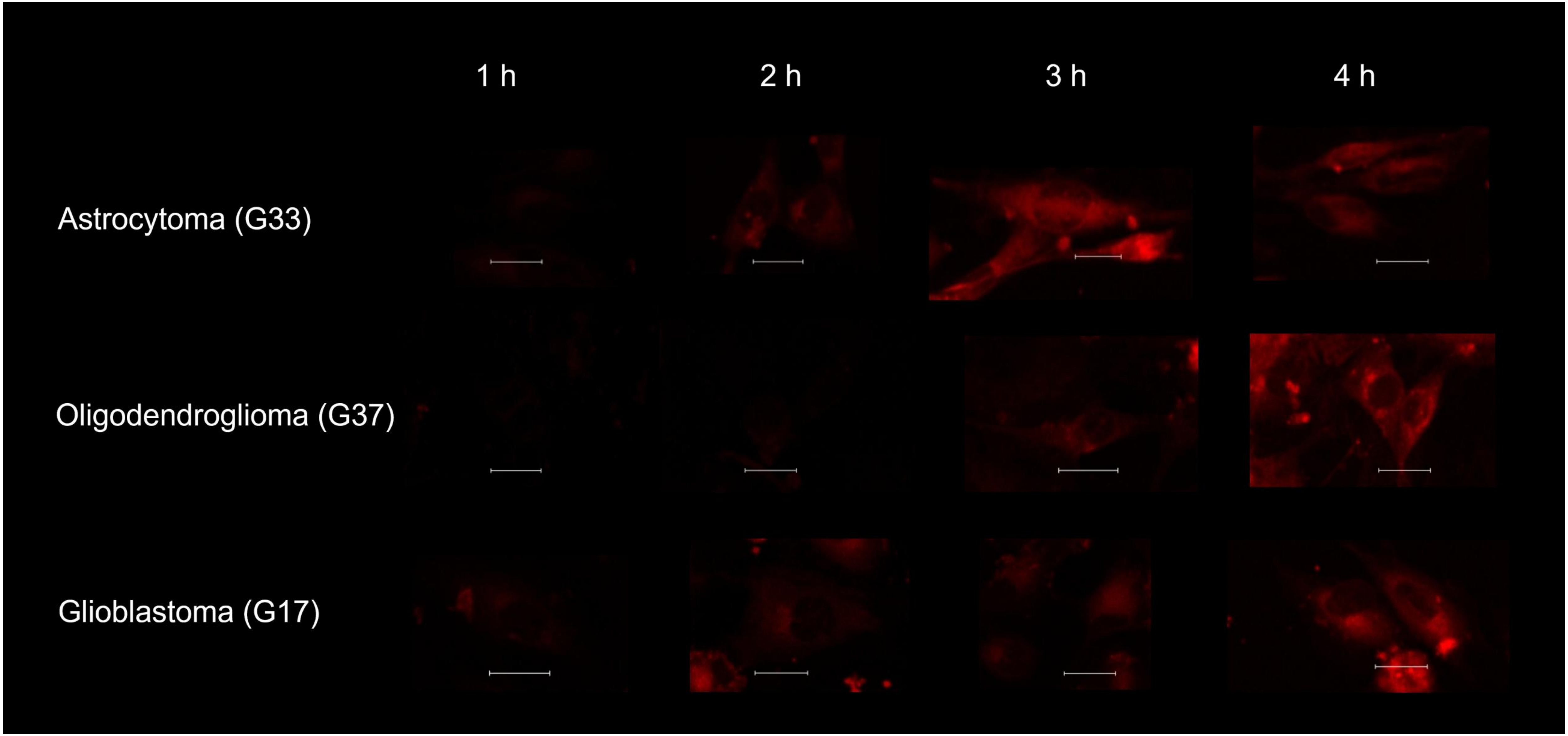

Supplementary figure 2. Dynamics of pz III accumulation in primary human glioma cell cultures. Representative confocal images of porphyrazine III accumulation in cultured astrocytoma (G33), oligodendroglioma (G55), and glioblastoma (G24). Uptake was analyzed during up to 4 hours of incubation with pz III (3 μM). Images were acquired at λex 561 nm and λem 600–700 nm; scale bars: 20 μm.

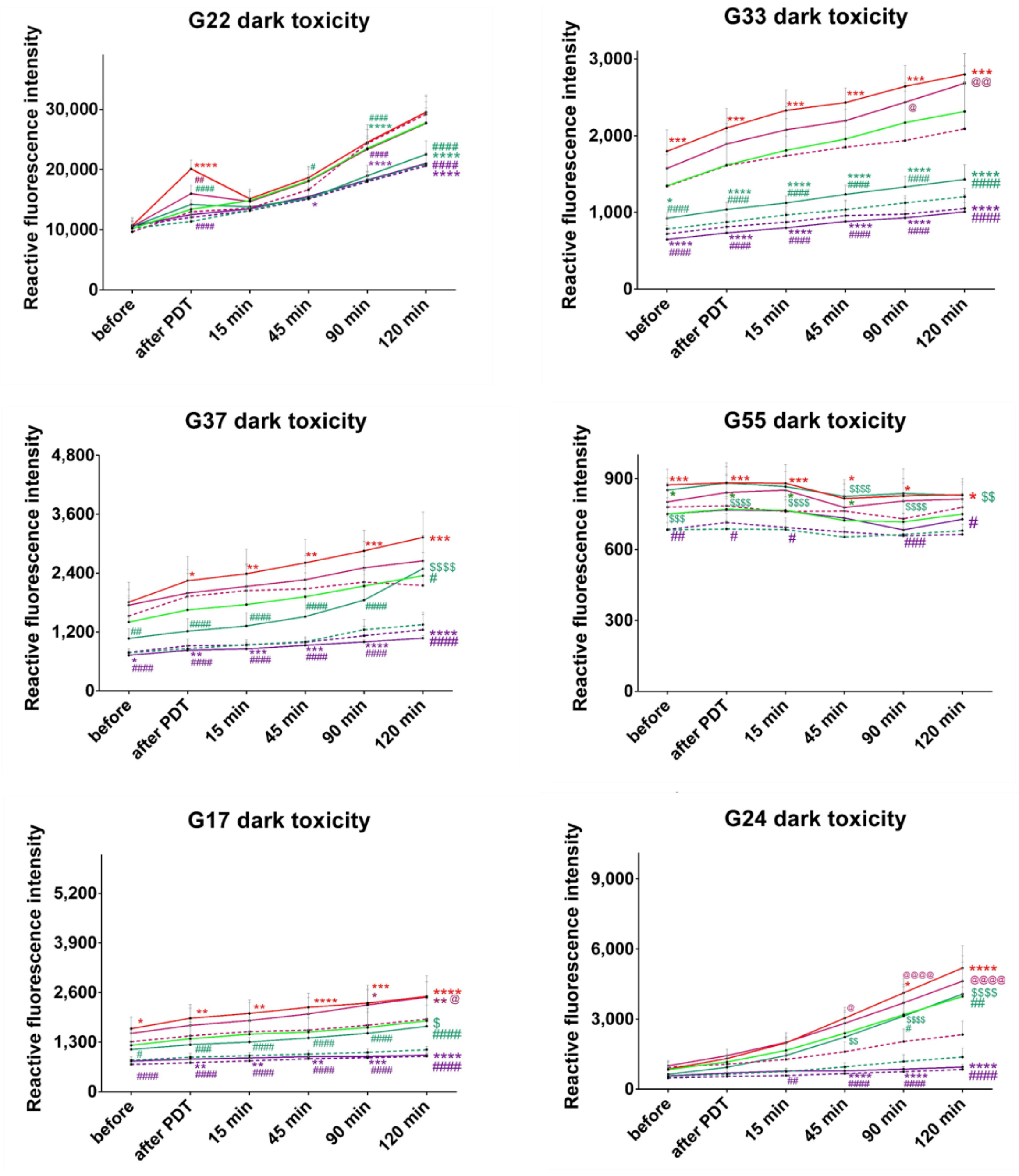

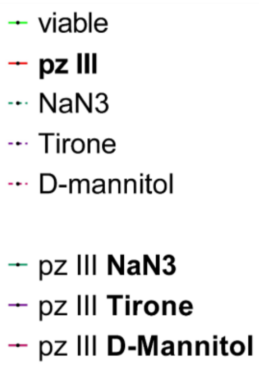

Supplementary figure 3. Determination of the contribution of various ROS forms to oxidative stress in pz III-loaded glioma cells using selective inhibitors and the DCFH-DA probe. Cells were incubated for 20 hours with selective ROS inhibitors at a concentration of 10 mM: NaN_3 for singlet oxygen ($^1O_2$), D-mannitol for hydroxyl radical (•OH), and Tiron for superoxide ($O_2•^-$). Then, cells were incubated in serum-free medium simultaneously with pz III and the inhibitors for 4 hours. After the loading step, DCFH-DA staining was performed (10 μM, 30 min) in PBS. Following incubation, the medium was replaced with PBS, and fluorescence was recorded (λex 488 nm, λem 525 nm). Fluorescence was measured over a period of 120 minutes after changing the medium. Mean values ± SD are presented, $n \geq 6$. The analysis was performed using two-way ANOVA with Tukey's multiple comparisons test. *— difference from the viable group (cells not subjected to PDT treatment; basal ROS levels in the culture were determined using DCFH-DA). #—difference from the pz III group (cells subjected to pz III-PDT; DCFH-DA detects the entire pool of ROS produced as a result of the treatment). $, &, and @—differences from the NaN3, Tiron, and D-mannitol groups, respectively. *, #, $, &, @ - $p < 0.05$, **, ##, $$, &&, @@ —$p < 0.01$, ***, ###, $$$, &&&, @@@ —$p < 0.001$, ****, ####, $$$$, &&&&, @@@@ —$p < 0.0001$.

Supplementary table 1. Statistical analysis of differences between groups in the experiment evaluating the type of ROS produced by primary cultures following photodynamic treatment using porphyrazine III (pz III). The analysis was performed using two-way ANOVA with Tukey's multiple comparisons test.

*— difference from the viable group (cells not subjected to PDT treatment; basal ROS levels in the culture were determined using DCFH-DA). #[M1.1][TV1.2]—difference from the pz III group (cells subjected to pz III-PDT; DCFH-DA detects the entire pool of ROS produced as a result of the treatment). $[M2.1][TV2.2], &[M3.1][TV3.2], and @[M4.1][TV4.2]—differences from the NaN3, Tiron, and D-mannitol groups, respectively. *, #, $, &, @ - $p < 0.05$, **, ##, $$, &&, @@ —$p < 0.01$, ***, ###, $$$, &&&, @@@ —$p < 0.001$, ****, ####, $$$$, &&&&, @@@@ —$p < 0.0001$.

	comparison groups	viable vs pz III	viable vs NaN_3	viable vs Tiron	vable vs D-Manitol	viable vs NaN_3 pz III	viable vs Tiron pz III	vable vs D-Manitol pz III	NaN_3 vs NaN_3 pz III	Tiron vs Tiron pz III	D-Manitol vs D-Manitol pz III	pz III vs NaN_3 pz III	pz III vs Tiron pz III	pz III vs D-Manitol pz III
G17 PDT	before	**											##	
	after	****						****			@@@@	####	####	
	15 min	****			****				$		@@@@	####	####	
	45 min	****						****	$		@@@@	####	####	
	90 min	****						****	$$		@@@@	####	####	####
	120 min	****						****	$$		@@@@	####	####	
G17 dark toxicity	before	*										#	####	
	after	**		**			*					###	####	
	15 min	**	*	**			**					####	####	
	45 min	****	*	**			**					####	####	
	90 min	***	**	***			***	*				####	####	
	120 min	****	***	****			****	**	$		@	####	####	
G22 PDT	before													
	after													
	15 min	****	*					***	$$		@@	####	####	####
	45 min	****	****	****			**	*	$$		@@	####	####	####
	90 min	****	****	****		*	****		$$		@@	####	####	####
	120 min	****	****	****		***	****	*	$$$		@@	####	####	####
G22 dark toxicity	before													
	after	****										####	####	##
	15 min													
	45 min			*		*						#		
	90 min		****	****		****	****					####	####	
	120 min		****	****		****	****					####	####	
G24 PDT	before													
	after	****				****		****	$$$$		@@@@		####	
	15 min	****				****		****	$$$$		@@@@	#	####	
	45 min	****				****		****	$$$$		@@@@	#	####	
	90 min	****				****		****	$$$$		@@@@	#	####	
	120 min	****				****		****	$$$$		@@@@	#	####	

G24 dark toxicity	**before**													
	**after**													
	**15 min**		*	**									##	
	**45 min**		****	****			****		$$		@		####	
	**90 min**	*	****	****	**		****		$$$$		@@@@	#	####	
	**120 min**	****	****	****	****		****		$$$$		@@@@	##	####	
G33 PDT	**before**	**						*					##	
	**after**	**						*					##	
	**15 min**	****						***			@@		####	
	**45 min**	****				*		****	$$		@@@		####	
	**90 min**	****				**		****	$$		@@		####	
	**120 min**	****				**		****	$$		@		####	
G33 dark toxicity	**before**	****	***	****		*	****					####	####	
	**after**	***	****	****		***	****					####	####	
	**15 min**	****	****	****		****	****					####	####	
	**45 min**	***	****	****		****	****					####	####	
	**90 min**	***	****	****		****	****				@	####	####	
	**120 min**	***	****	****		****	****				**	####	####	
G37 PDT	**before**		*	*			*					#	##	
	**after**	****	**	**								####	####	
	**15 min**	****	***	***			*	*			@	####	####	
	**45 min**	****	***	***			**	**			@	####	####	
	**90 min**	****	****	****			**	****				####	####	
	**120 min**	****	***	****			**	**				####	####	
G37 dark toxicity	**before**						*					##	####	
	**after**	*	**	*			**					####	####	
	**15 min**	**	**	**			***					####	####	
	**45 min**	**	***	***			***					####	####	
	**90 min**	***	**	****			****					####	####	
	**120 min**	***	***	****			****		$$$$			#	####	
G55 PDT	**before**													
	**after**													
	**15 min**													

	**45 min**													
	**90 min**													
	**120 min**													
G55 dark toxicity	**before**	***				*			$$$				##	
	**after**	***				*			$$$$				#	
	**15 min**	***				*			$$$$				#	
	**45 min**	*				*			$$$$					
	**90 min**	*				*			$$$$				###	
	**120 min**	*							$$				#	
